# The nuclear and cytoplasmic roles of miR-320 in non-alcoholic fatty liver disease

**DOI:** 10.18632/aging.104040

**Published:** 2020-11-07

**Authors:** Jiabing Zhan, Huizhen Lv, Beibei Dai, Shuai Yuan, Jiahui Fan, Yanru Zhao, Zhongwei Yin, Dao Wen Wang, Chen Chen, Huaping Li

**Affiliations:** 1 Division of Cardiology, Department of Internal Medicine, Tongji Hospital, Tongji Medical College, Huazhong University of Science and Technology, Wuhan, China; 2 Hubei Key Laboratory of Genetics and Molecular Mechanisms of Cardiological Disorders, Wuhan, China; 3 State Key Laboratory of Experimental Hematology, National Clinical Research Center for Blood Diseases, Institute of Hematology and Blood Diseases Hospital, Chinese Academy of Medical Sciences and Peking Union Medical College, Tianjin, China; 4 Collaborative Innovation Center of Tianjin for Medical Epigenetics and Department of Physiology and Pathophysiology, Tianjin Medical University, Tianjin Key Laboratory of Metabolic Diseases, Tianjin, China

**Keywords:** miR-320, cytoplasm, nuclear, metabolic disorders, hyperlipidemia

## Abstract

Background: Non-alcoholic fatty liver disease (NAFLD) is the most common chronic liver disorder worldwide. Multiple metabolic disorders, such as hyperlipidemia, hyperglycemia, insulin resistance and obesity, have been reportedly associated with NAFLD, but little is known about the detailed mechanisms.

Methods and Results: Here, we explored the effects of multiple metabolic disorders, especially hyperglycemia on lipid accumulation in liver using several well-established animal models. We found that liver lipid deposition was increased in both type 1 diabetes and high-fat diet (HFD) induced hyperlipidemia models, suggesting that either hyperglycemia or hyperlipidemia alone or together was able to trigger NAFLD. Moreover, we tested whether miR-320, a miRNA promoting lipid accumulation in heart revealed by our previous study, also participated in NAFLD. Though miR-320 treatment further increased liver lipid deposition in type 1 diabetes and HFD-feeding mice, it showed no effect in leptin-receptor deficient db/db mice. Interestingly, miR-320 affected different target genes in cytosol and nucleus, respectively, which collectively led to liver lipid overload.

Conclusions: Our findings illustrated the complex roles of miRNAs in subcellular fractions including nucleus and cytoplasm, which may lead to new insights into the mechanisms and treatment strategies for NAFLD in the future.

## INTRODUCTION

Nonalcoholic fatty liver disease (NAFLD) is the most common chronic liver disorder worldwide, affecting one in four adults, with the global prevalence between 22.10% to 28.65% [1]. Although the frequency of its liver related mortality is low, the staggering denominator of over a billion adults with NAFLD coupled with a ~2% lifetime risk of liver related mortality will eventually lead to ~20,000,000 liver related deaths among patients currently alive with NAFLD [2]. NAFLD is becoming the most common cause of liver disease and liver related death globally [1]. However, no proven effective medial therapy is available for NAFLD currently [3].

NAFLD is defined by the accumulation of fat in the liver, with more than 5% of hepatocytes containing visible intracellular triglyceride, in the absence of excessive alcohol consumption and other causes of hepatic steatosis [4]. NAFLD is closely associated with metabolic co-morbidities including obesity, insulin resistance and dyslipidemia. Specifically, more than three-quarters of type 2 diabetes patients were reported having NAFLD [5, 6]. It is now clear that liver steatosis is closely linked to impaired insulin sensitivity and type 2 diabetes. Interestingly, previous studies suggested that hepatic steatosis preceded the development of hepatic and adipose tissue insulin resistance, as well as systemic hyperglycemia and hyperinsulinemia in mice fed with high-fat diet (HFD) [7, 8]. Notably, changes occurring in the fatty liver of NAFLD could cause insulin resistance by altering paracrine and endocrine functions, which in turn would accelerate the development of NAFLD itself through impaired ability of insulin to suppress adipose tissue lipolysis, increased delivery of free fatty acids to the liver, and increased de novo lipogenesis [9], eventually triggering a vicious cycle between NAFLD and metabolic co-morbidities [10]*.*

While the roles of HFD and type 2 diabetes in NAFLD are becoming clear, few data are available regarding the prevalence and natural history of NAFLD in people with type 1 diabetes [11]. In animal studies, NAFLD was usually induced by specific diet (e.g. HFD) or genetic manipulation (e.g. db/db or ob/ob) [12]. Because multiple metabolic disorders, such as hyperglycemia, hyperlipemia and insulin resistance, were observed in these animals simultaneously, the specific effects of hyperglycemia alone on NAFLD is unclear. Therefore, in this study, we take advantage of a well-established animal model, the streptozotocin (STZ)-induced type 1 diabetic model, in comparison with the HFD-induced hyperlipidemia model, and db/db (leptin receptor deficient) type 2 diabetes model, to explore the certain effects of each metabolic disorder, especially the effects of hyperglycemia alone on NAFLD.

MiRNAs are recently discovered small (~22 nt) non-coding RNAs that usually negatively regulate gene expression at post-transcriptional levels in various biological processes. We have previously demonstrated that miR-320 overexpression could increase circulating triglyceride and cholesterol levels in ApoE^−/−^ mice [13], and moreover, miR-320 overexpression promoted while miR-320 inhibition rescued aortic lipid accumulation in ApoE^−/−^ mice [13]. Interestingly, we subsequently found that miR-320 was also presented in the nucleus to enhance CD36 transcription and induce fatty acids accumulation in diabetic db/db mice [14]. Importantly, miR-320 inhibition was able to prevent cardiac lipid accumulation in db/db mice [14]. Our previous data strongly suggested that miR-320 was a potent regulator in multiple metabolic disorders, but its role in NAFLD was largely unknown.

In the current study, we evaluated the effects of metabolic disorders, especially the effects of hyperglycemia alone on NAFLD. Furthermore, we investigated the effects of miR-320 on liver lipid accumulation and whether it could serve as a potential therapeutic target for NAFLD in three different animal models. Finally, we identified the direct targets of miR-320 and evaluated the subcellular regulation pattern in hepatocytes.

## RESULTS

### Lipid content in the liver of differently treated mice

To explore the effects of single metabolic disorder on NAFLD, we utilized three animal models including the STZ-induced type 1 diabetes model, HFD-induced obesity model and the db/db type 2 diabetes model (Figure 1A). In STZ-treated C57BL/6 mice, previous studies have demonstrated that as early as 4-5 weeks after STZ administration, only hyperglycemia was observed. Later, hyperlipidemia will be induced 5-17 weeks after STZ administration [15]. To investigate the effect of hyperglycemia alone on NAFLD, in this study, we analyzed the circulating biochemical parameters and liver lipid levels. As expected, 4 weeks after STZ treatment, only fasting blood glucose (FBG) was increased, while circulating triglyceride (TG) and total cholesterol (TC) remained unchanged (Figure 1B).

**Figure 1:**
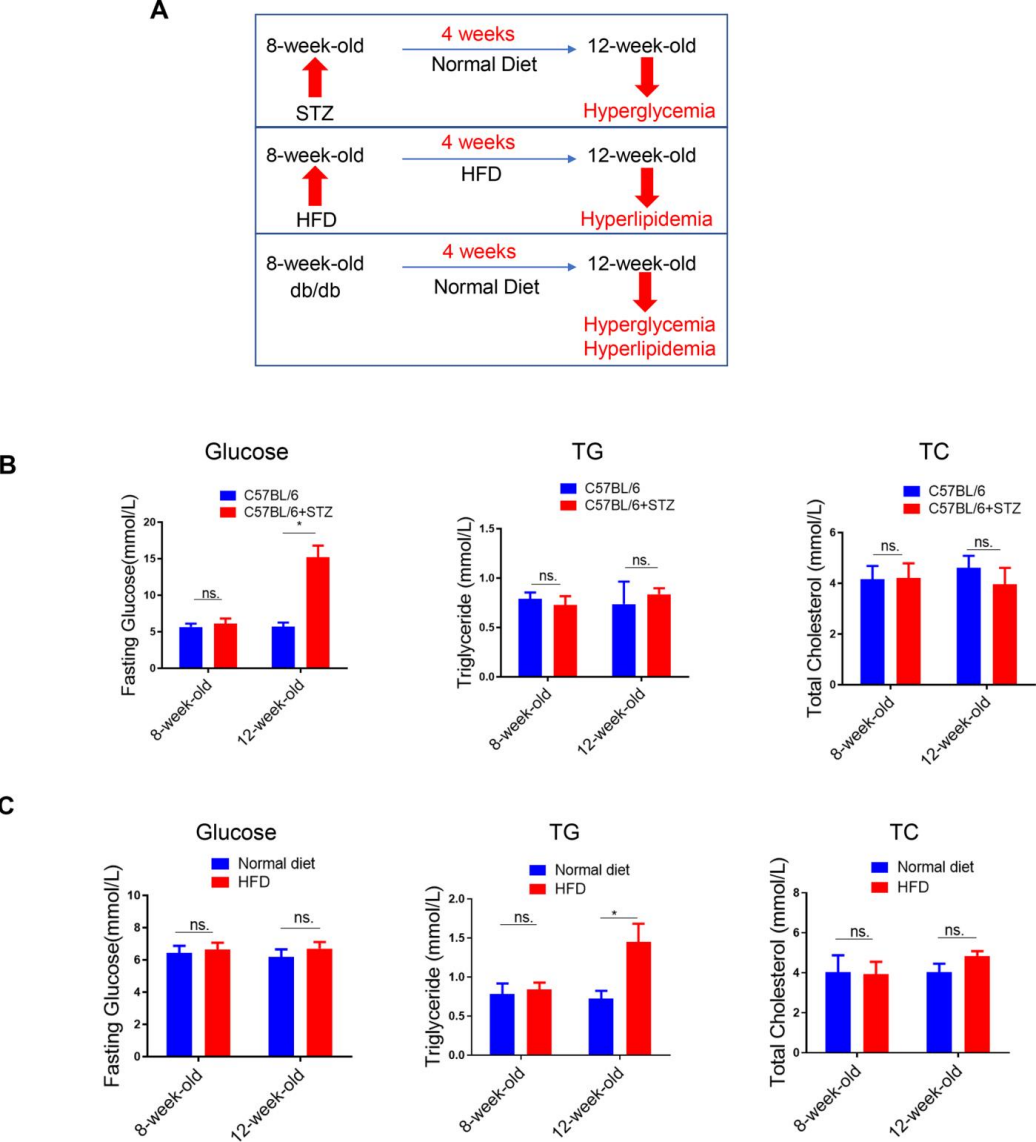
**Lipid content in the liver of differently treated mice.** (**A**) Summarized data of the effects of short-term (4 weeks) STZ-, HFD-, LEPR-deficiency (db/db) treatments on circulating glucose and lipid levels. (**B**) Time course analysis of circulating glucose, TG, TC levels in STZ-treated C57BL/6 mice and control mice (n=3-4, *p<0.05). (**C**) Time course analysis of circulating glucose, TG, TC levels in HFD-treated C57BL/6 mice and control mice (n=3-4, *p<0.05).

**Figure 1:**
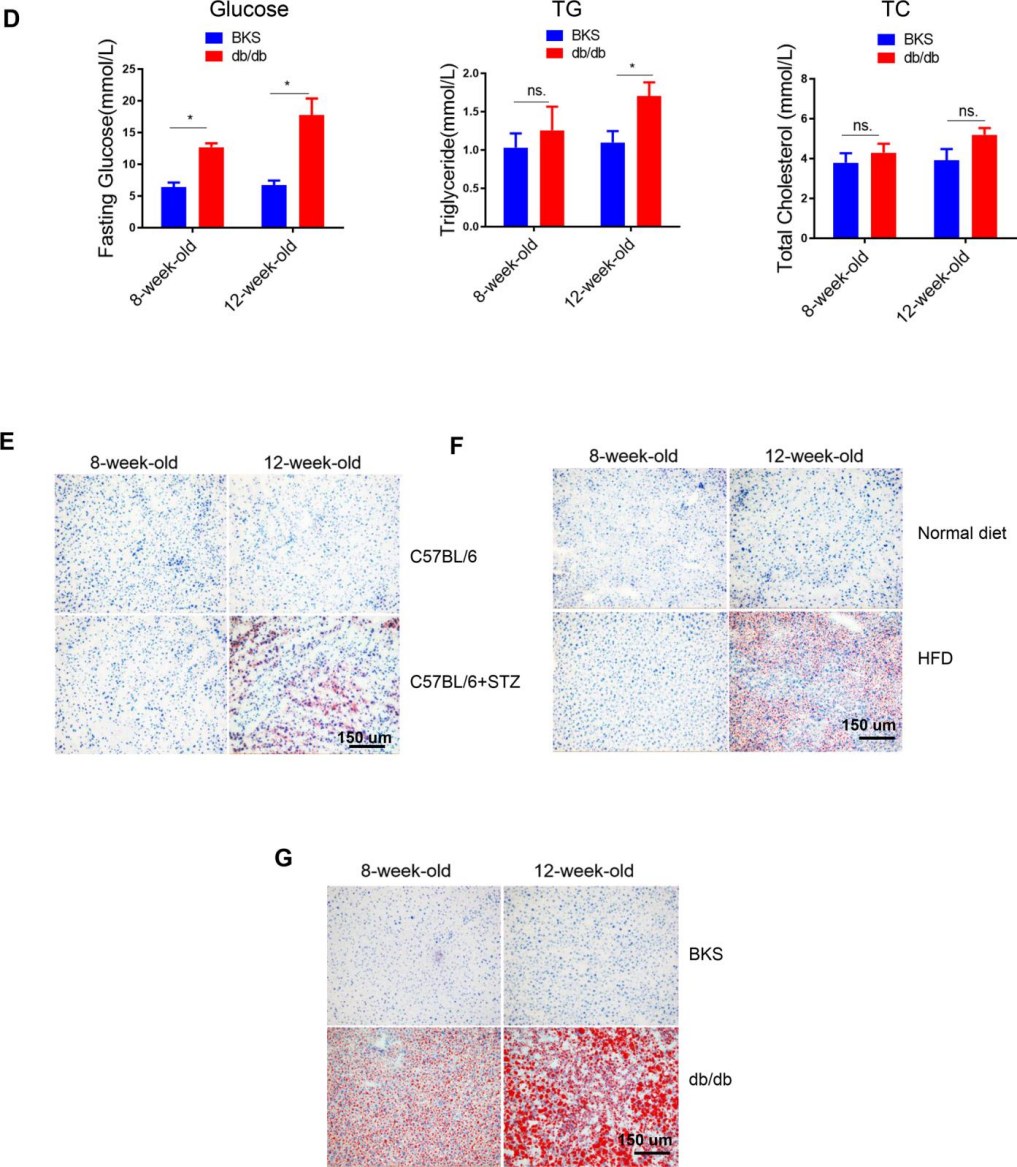
**Lipid content in the liver of differently treated mice.** (**D**) Time course analysis of circulating glucose, TG, TC levels in db/db mice in comparison with wild type mice (n=3-4, *p<0.05). (**E**–**G**) Representative image of liver lipid contents detected by oil-red staining in STZ-treated C57BL/6 mice (**E**), HFD-treated C57BL/6 mice (**F**) and db/db mice (**G**).

We also analyzed the same parameters in HFD-treated C57BL/6 mice. Previously, we have shown that blood glucose level was increased in 8-week-HFD treated mice but not in 4-week-HFD treated mice [14]. Therefore, in the current study, we treated mice with HFD for 4 weeks to explore the effect of hyperlipidemia alone on NAFLD. In contrast to the 4 weeks STZ treatment, 4 weeks HFD treatment led to upregulated TG but unchanged FBG and TC (Figure 1C).

In the third model, the leptin receptor (LEPR) deficient db/db mice, hyperglycaemia combined with hyperlipidemia were observed in 12-week-old db/db mice, compared to wild type mice (Figure 1D).

Meanwhile, oil-red staining revealed that liver lipid levels were increased at 12-week-old in all the three models (Figure 1E–1G). HE staining also showed clear steatosis at 12-week-old in these animal models (Supplementary Figure 2). These data suggested that either STZ-induced hyperglycaemia or HFD-induced hyperlipidemia alone was sufficient to trigger increased lipid deposition in liver, respectively.

### Fatty acids uptake and lipogenesis in liver of differently treated mice

**Figure 2:**
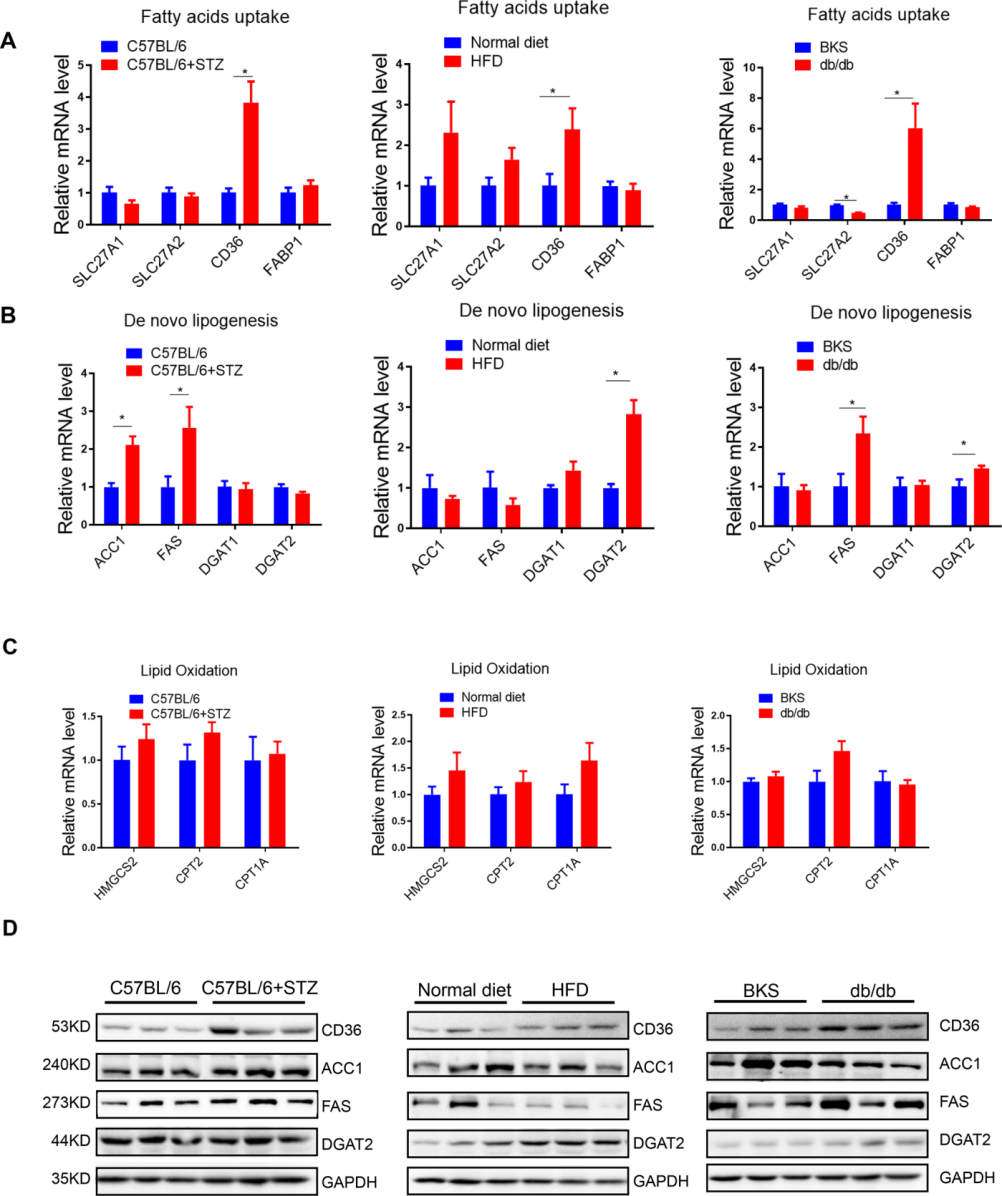
**Fatty acids uptake and lipogenesis in liver of differently treated mice.** (**A**–**C**) Quantitative real-time PCR was performed to determine the hepatic mRNA levels of fatty acids uptake-, de novo lipogenesis- and lipid oxidation related genes in STZ-treated C57BL/6 mice, HFD-treated C57BL/6 mice and db/db mice (n=6, *p<0.05). (**D**) Western blot was used to analyse the hepatic protein levels of fatty acids uptake-, de novo lipogenesis- and lipid oxidation related genes in STZ-treated C57BL/6 mice, HFD-treated C57BL/6 mice and db/db mice (n=3).

To explore the mechanism underlying increased liver lipid deposition in differently treated mice, we analysed critical gene markers for fatty acids uptake, lipogenesis and lipid oxidation processes by qRT-PCR. Interestingly, we found that among the four fatty acids uptake relative genes, only fatty acid translocase (FAT)/CD36 was significantly increased in all three animal models (Figure 2A), indicating that hyperglycaemia alone or hyperlipidemia alone could increase fatty acids transport into hepatocytes.

In terms of lipogenesis, we detected lipogenic enzymes Acetyl-CoA carboxylase 1 (ACC1, a biotin-containing enzyme which catalyzes the carboxylation of acetyl-CoA to malonyl-CoA, the rate-limiting step in fatty acid synthesis), Fatty Acid Synthase (FAS, catalyzes the conversion of acetyl-CoA and malonyl-CoA to the 16-carbon fatty acid palmitate), Diacylglycerol O-Acyltransferase 1 (DGAT1, a key enzyme to catalyze the conversion of diacylglycerol and fatty acyl CoA to triacylglycerol) and DGAT2 (catalyzes the terminal and only committed step in triacylglycerol synthesis). Interestingly, we found that ACC1 and FAS were increased by STZ treatment, while DGAT2 was upregulated in HFD-treated mice (Figure 2B), indicating that hyperglycemia and hyperlipidemia might promote lipogenesis by activating distinct lipogenic genes, respectively. Moreover, we noted that FAS, but not ACC1, was increased in both type 1 (STZ induced) and type 2 (db/db) diabetes model, indicating that lipogenic enzyme FAS might be specifically induced by hyperglycemia.

In terms of lipid oxidative genes, including HMGCS2, CPT2 and CPT1A, no significant changes were observed (Figure 2C), indicating that lipid oxidation might not be responsible for the increased lipid deposition in liver, at least not in the early stages of hyperglycaemia or hyperlipidemia induced NAFLD.

Notably, protein levels of these crucial genes showed consistent regulation patterns as mRNA levels in these animal models (Figure 2D).

### MiR-320 overexpression increased liver lipid content in differently treated mice

Previously, we have shown that miR-320, a small non-coding RNA molecule, promoted lipid accumulation in aorta and heart by targeting SRF and CD36, respectively, while miR-320 inhibition rescued lipid overload in these tissues [13, 14]. We then tested whether miR-320 was participated in the increased liver lipid deposition. Although we found that the global miR-320 level in liver was unchanged in these animal models (Figure 3A), as our previous work and other studies have suggested the distinct roles of one certain miRNA in different subcellular fractions [16, 17], we then measured the subcellular localization of miR-320 in liver of these animal models, finding that miR-320 was similarly expressed in cytoplasm and nucleus in mouse liver under normal condition (Figure 3B). Interestingly, increased nuclear miR-320 was observed in livers from STZ-treated, HFD-treated C57BL/6 mice and db/db mice in comparison with control mice, respectively (Figure 3C). While in cytosol, miR-320 levels showed a decreasing trend in these animal models (Figure 3D). These data indicated that miR-320 might translocate from cytoplasm into nucleus in NAFLD.

**Figure 3:**
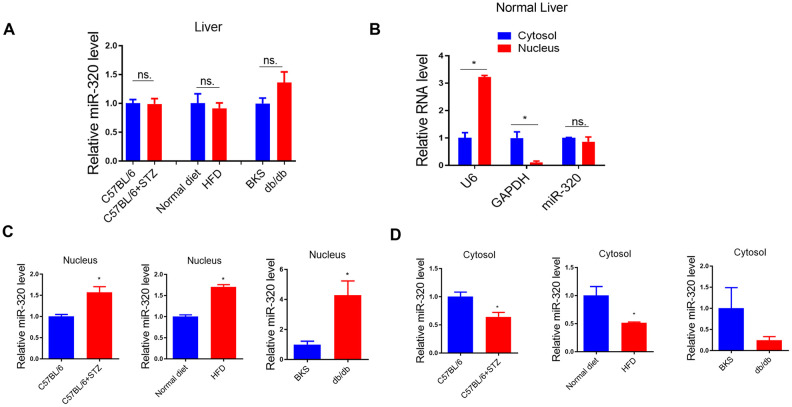
**MiR-320 overexpression increased liver lipid content in differently treated mice.** (**A**) MiR-320 levels in STZ-treated C57BL/6 mice, HFD-treated C57BL/6 mice, and db/db mice liver were determined by quantitative real-time PCR (n=6). (**B**) Cytoplasmic and nuclear miR-320 levels in normal mice liver were detected by cell fractionation followed by RT-qPCR. GAPDH mRNA and U6 RNA were served as cytoplasmic and nuclear markers. MiR-320 was similarly expressed in nucleus and cytoplasm (n=3, *p<0.05). (**C**, **D**) Cytoplasmic and nuclear miR-320 levels in STZ-treated C57BL/6 mice (**B**), HFD-treated C57BL/6 mice (**C**), and db/db mice (**D**) liver were determined using cell fractionation followed by quantitative real-time PCR (n=3, *p<0.05).

**Figure 3:**
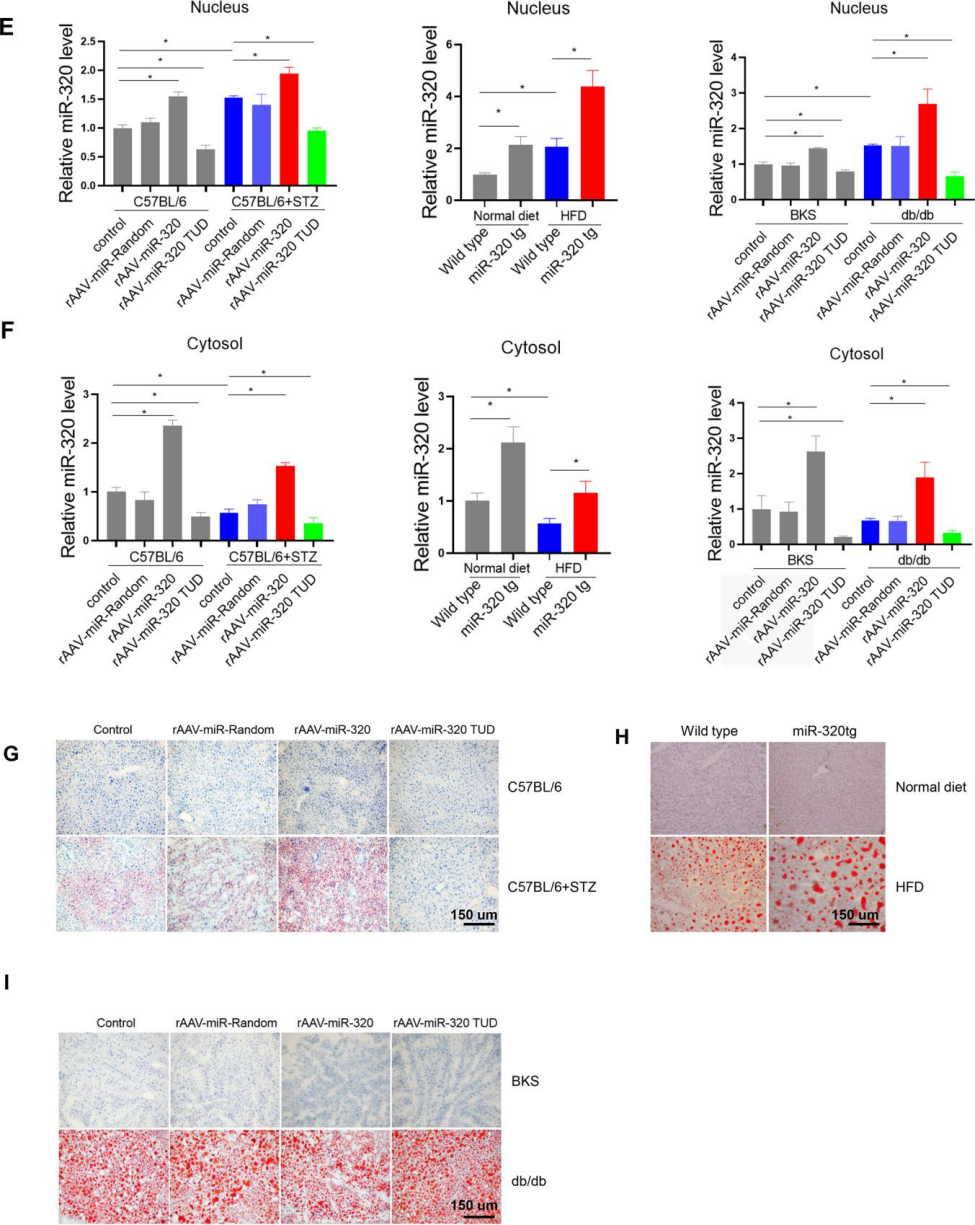
**MiR-320 overexpression increased liver lipid content in differently treated mice.** (**E**, **F**) Nuclear and cytosol miR-320 levels in miR-320 transgenic and rAAV-miR-320 treated mice were detected by quantitative real-time PCR (n=3, *p<0.05). (**G**) The effects of rAAV-miR-320 on lipid accumulation in liver determined by oil red staining in STZ-treated C57BL/6 mice. (**H**) Detection of lipid levels by oil red staining in miR-320 transgenic mice treated with HFD. (**I**) The effects of rAAV-miR-320 on lipid accumulation in liver determined by oil red staining in db/db mice.

A step further, we tested whether overexpression or inhibition of miR-320 would change the lipid content in liver of these animal models. Successful induction or suppression of miR-320 in liver was introduced by using rAAV system or miR-320 transgenic mice (Figure 3E, 3F). We found that miR-320 overexpression increased STZ-induced lipid deposition in C57BL/6 mice while miR-320 inhibition reduced lipid content in liver (Figure 3G and Supplementary Figure 3A, 3D). Consistently, lipid acumination was more abundant in miR-320 transgenic mice fed with HFD compared to their wild type counterparts (Figure 3H and Supplementary Figure 3B, 3D). These data indicated that miR-320 overexpression was able to further promote hyperglycemia or hyperlipidemia induced lipid deposition, respectively, and miR-320 inhibition might be a therapeutic strategy for NAFLD. In normal unstressed mice, neither miR-320 overexpression nor inhibition had any detectable effect on lipid acumination in liver (Figure 3G, 3H). On the contrary, we found that miR-320 had no effect on lipid accumulation in db/db mice liver (Figure 3I and Supplementary Figure 3C, 3D), indicating that miR-320 exhibited different regulation pattern in LEPR deficient db/db mice. In terms of circulating glucose, TG and TC, miR-320 overexpression or inhibition did not affect these parameters in all three animal models (Supplementary Figure 4).

Moreover, we took advantage of AAV8-TBG system to specifically investigate the role of miR-320 in liver. These data showed that AAV8-TBG-miR-320 treatment exclusively increased hepatic miR-320 level, and promoted lipid deposition in STZ-induced and HFD-treated mice but not in db/db mice, which were consistent with the observations in global rAAV9 treatment (Supplementary Figure 5). These data suggested that miR-320 overexpression or knockdown, whether by using liver specific AAV8-TBG vector or global AAV9-CMV-vector, were both able to affect the outcomes of NAFLD.

### The regulation of miR-320 on lipogenesis was leptin-receptor dependent

We further investigated the mechanism underlying the different effects of miR-320 on lipid content between STZ or HFD-treated mice and the LEPR deficient db/db mice. Coincidently, by bioinformatic prediction, we found that LEPR 3’ UTR harbored a miR-320 binding site, which was conserved among human and mouse (Figure 4A). We then tested whether miR-320 regulated liver lipid metabolism through leptin receptor pathway. We found that miR-320 indeed decreased LEPR, but not leptin in human hepatic L02 cells (Figure 4B). We then evaluated the effects of miR-320 on lipid metabolism related gene in LEPR-knockdown L02 cells. Interestingly, we found that LEPR-knockdown completely blocked miR-320 induced FAS activation and partly blocked CD36 activation (Figure 4C). These data suggested that the regulation of miR-320 on lipogenesis gene FAS might be leptin-receptor dependent, while the regulation of miR-320 on fatty acids uptake gene CD36 was partly leptin-receptor dependent.

**Figure 4:**
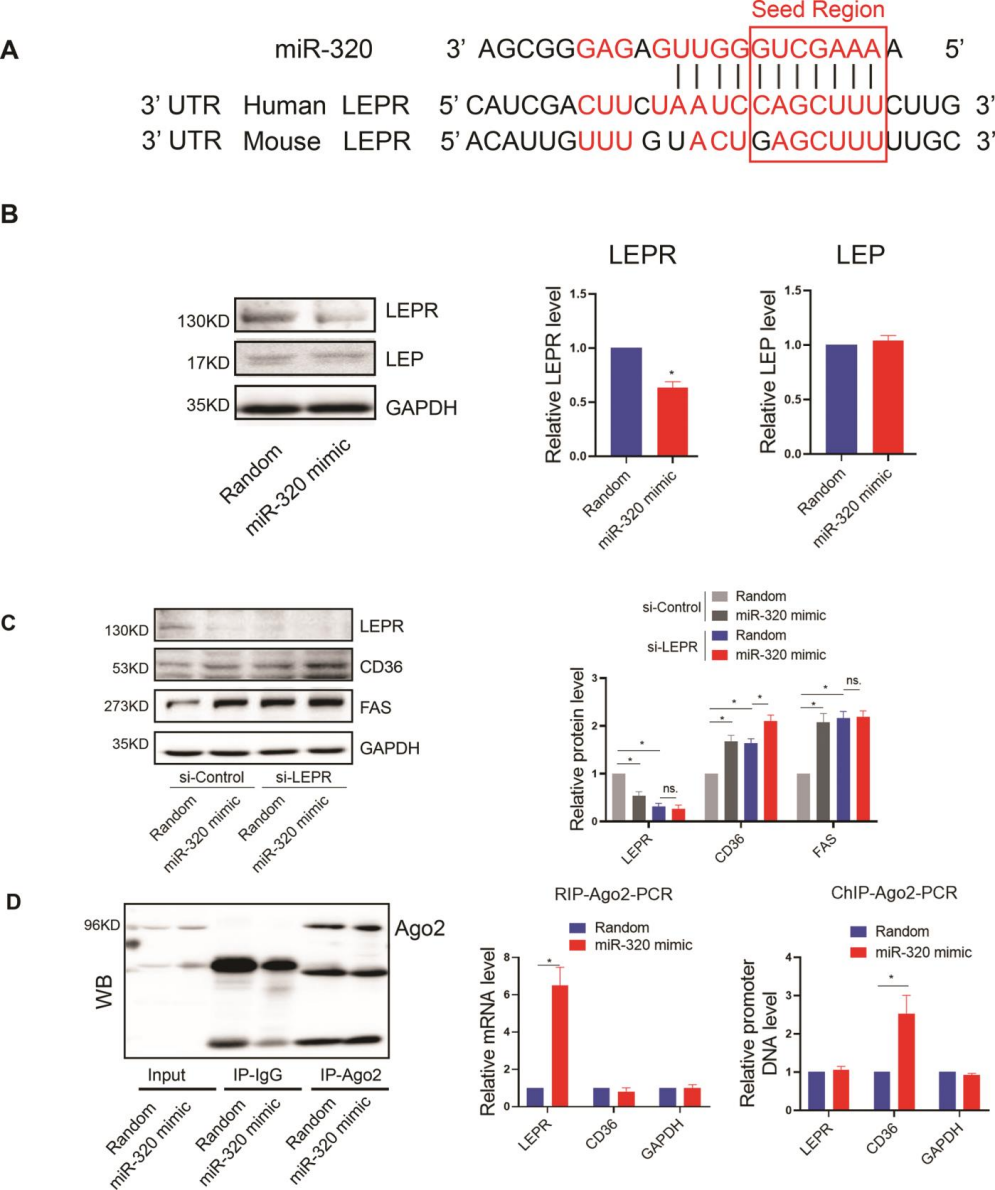
**The regulation of miR-320 on lipogenesis was leptin-receptor dependent.** (**A**) Sequence alignment of miR-320 on 3’ UTR of LEPR from mouse and human. (**B**) Western blot was performed to determine the protein levels of LEP and LEPR in L02 cells treated with miR-320 mimic (n=3, *p<0.05). (**C**) Effects of miR-320 mimic on protein levels of CD36, FAS and DGAT2 in si-NC and si-LEPR treated L02 cells detected By Western blot (n=3, *p<0.05). (**D**) Relative mRNA levels and promoter DNA levels of LEPR and CD36 detected by Ago2-RIP and Ago-2 CHIP, respectively (n=3, *p<0.05).

**Figure 4:**
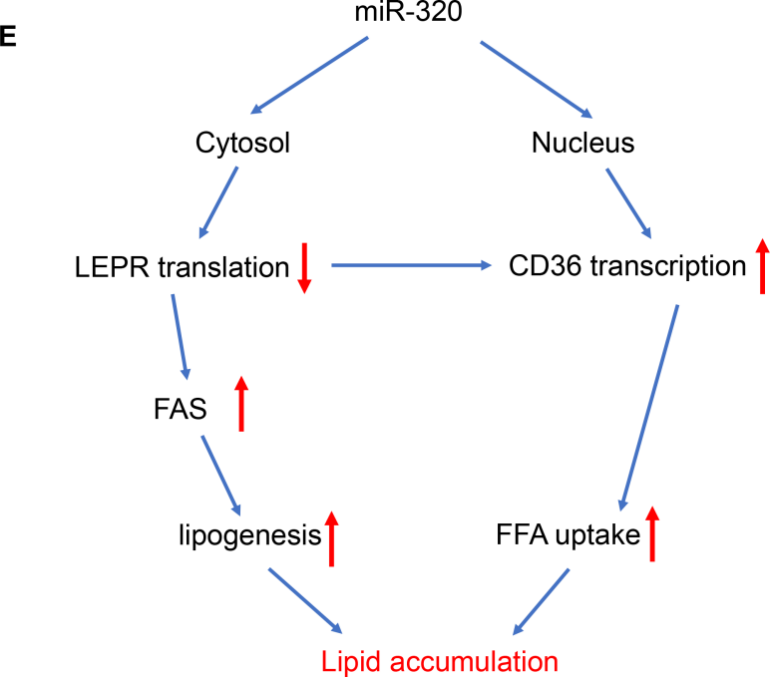
**The regulation of miR-320 on lipogenesis was leptin-receptor dependent.** (**E**) A model to illustrate the effects of cytosol and nuclear miR-320 on lipid accumulation.

In terms of fatty acids uptake gene CD36, we have previously demonstrated the direct activation of CD36 by nuclear miR-320 in cardiomyocytes [14], we then tested whether this is also the case for nuclear and cytosol miR-320 in hepatocytes. As miRNAs regulate gene expression through Ago2, a key protein binds microRNAs and mediates the loading of these small noncoding RNAs onto RISC to recognize specific targets through base-pairing [18]. We therefore performed RNA co-immunoprecipitation and Chromatin Immunoprecipitation with anti-Ago2. As a result, Ago2-CHIP and Ago2-RIP analysis further confirmed the direct binding of Ago2-miR-320 complex on promoter DNA of CD36 gene and 3’ UTR of LEPR mRNA, respectively (Figure 4D).

These data suggested that miR-320 directly targeted LEPR mRNA and CD36 DNA, respectively, to regulate these genes expression, indicating complicated roles of one certain miRNA in different subcellular fractions (Figure 4E).

### miR-320 targeted CD36 promoter DNA in nucleus and leptin receptor mRNA in cytoplasm respectively

**Figure 5:**
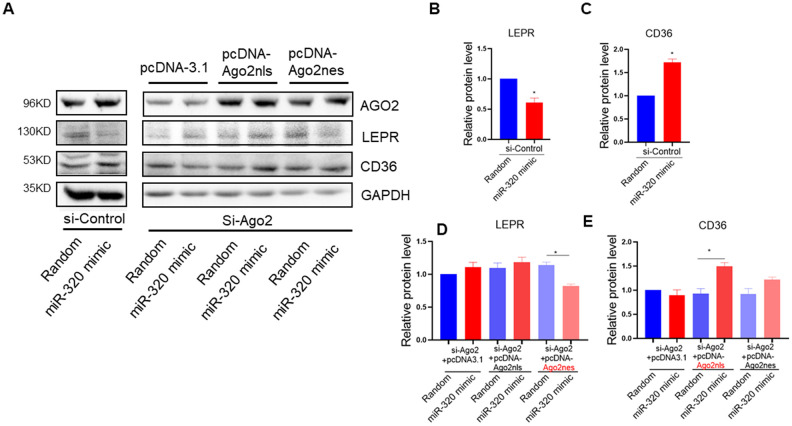
**miR-320 targeted CD36 promoter DNA in nucleus and leptin receptor mRNA in cytoplasm respectively.** (**A**–**E**) Western blotting analysis of the effects of miR-320 on CD36 and LEPR rescued with nuclear or cytosol Ago2 re-expression. In Ago2 knockdown cells, re-expression of Ago2 in the cytoplasm restored miR-320-mediated LEPR suppression. While re-expression of Ago2 in the nucleus restored miR-320-mediated CD36 activation (n=3, *p<0.05).

To answer the question whether the regulation of miR-320 on these lipid metabolic relative genes were mainly cytoplasmic or nuclear effects, we took advantage of Ago2 knockdown hepatocytes to functionally rescue miR-320-meidiated gene regulation with pcDNA-Ago2nes (nuclear export signal) targeting cytoplasm or pcDNA-Ago2nls carrying a nuclear targeting signal peptide, respectively. We found that miR-320 decreased LEPR and increased CD36 expression in control hepatocytes, while it lost these effects in Ago2 knockdown (siRNA treated) L02 cells (Figure 5A–5E). In Ago2 knockdown cells, re-expression of Ago2 in the cytoplasm restored miR-320-mediated LEPR suppression (Figure 5A, 5D). While re-expression of Ago2 in the nucleus restored miR-320-mediated CD36 activation (Figure 5A, 5E). In animal models, we also observed upregulation of CD36 and downregulation of LEPR by miR-320 overexpression (Supplementary Figure 6).

Collectively, these data demonstrated that miR-320 could directly target CD36 promoter DNA in nucleus and LEPR mRNA in cytoplasm to regulate their expression, respectively. Notably, miR-320 activated its nuclear target CD36 while suppressed its cytoplasmic target LEPR simultaneously, indicating the complicated and coordinated regulation pattern of miRNAs in diseases.

## DISCUSSION

In this study, we found that liver lipid deposition was increased in type 1 diabetes and HFD induced hyperlipidemia models, suggesting that hyperglycaemia alone or hyperlipidemia alone was able to trigger NAFLD. Moreover, we also tested whether miR-320, a miRNA we have previous shown to promote lipid accumulation in aorta and heart, was able to affect NAFLD. Interestingly, miR-320 treatment further increased liver lipid deposition in STZ-induced and HFD-treated mice but not in db/db mice, partly because miR-320 promoted lipogenesis through leptin-receptor signaling. Mechanically, miR-320 inhibition decreased miR-320 level in cytosol, increased leptin receptor translation and decreased de novo lipogenesis, meanwhile, miR-320 inhibitor also decreased miR-320 level in nucleus, reduced CD36 transcription and lipid uptake, which collectively leading to rescued liver lipid overload. These new mechanisms suggested a new potential therapy for treating NAFLD with miR-320 inhibitor and illustrated the complex roles of miRNAs in subcellular fractions including nucleus and cytoplasm, which may lead to new insights into the mechanisms and treatment strategies for NAFLD in the future.

Though the associations between NAFLD and metabolic co-morbidities such as diabetes and dyslipidemia are generally clear, the underlying mechanisms are largely unknown. Especially, whether hyperglycemia alone would lead to increased liver lipid accumulation is largely unclear. To explore whether hyperglycemia or dyslipidemia contributed to liver lipid deposition, we took advantage of STZ-induced and high fat feeding mice and evaluated liver lipids only 4-weeks after the treatment, which resulted in isolated hyperglycemia or dyslipidemia. Because a longer time treatment would lead to multiple metabolism disorders triggering the formation of a vicious cycle between hyperglycemia and dyslipidemia [14, 15], which would make it harder to determine the cause-and-effect factors for NAFLD. As for this reason, we only analyzed liver lipid deposition after 4-weeks STZ- or high fat diet treatment and observed only modest induction in liver lipid content. Notably, inflammation and fibrosis, the crucial indicators for non-alcoholic steatohepatitis (NASH) or cirrhosis, were undetectable yet in our study (data not shown). Here, because we only focused to study the early stage of fatty liver disease, the metabolic changes and the mechanisms underline the advanced or end-stage of NAFLD still await investigation.

We noted that miR-320 directly decreased LEPR translation in hepatocyte. Moreover, lipogenic gene FAS activation mediated by miR-320 was abolished by LEPR knockdown, suggesting that the effect of miR-320 on lipid deposition in liver was mainly through LEPR. This might explain why miR-320 promoted liver lipid deposition in STZ-induced and high fat feeding mice but not in db/db (leptin receptor deficient) mice. Very interestingly, we found that in db/db mice, miR-320 was actually able to promote lipid deposition in heart [14], which was very different to liver. We reasoned that this might because lipid accumulation in heart was mainly attributed by lipid uptake while in liver was contributed by lipogenesis and lipid uptake [19, 20]. Because lipogenic genes were regulated by LEPR, this might explain why miR-320 mediated lipogenesis was abolished in LEPR deficient db/db mice liver, while in STZ- and HFD mice (LEPR pathway and lipogenesis were preserved), miR-320 was able to promote lipid accumulation by enhancing both the lipogenesis and lipid uptake processes. In contrast, in db/db heart, miR-320 continued to enhance cardiac lipid accumulation by directly activating CD36 transcription through an LEPR in-dependent manner.

Back to these animal models, we did have observed upregulation of CD36 in liver of these mice (Figure 2D), which was consistent with increased nuclear miR-320 level (Figure 3C). Interestingly, increased hepatic LEPR level was also observed (Supplementary Figure 6), which was consistent with decreased miR-320 expression in cytoplasm (Figure 3D). Because LEPR loss of function led to liver lipid overload in db/db mice. The increase of LEPR in STZ- treated mice seemed to be a compensatory mechanism by which maintains the stability of the lipid metabolism. However, due to other pro-lipogenic factors induced by hyperglycemia, the overall lipogenic gene (FAS) in STZ- treated mice was still increased. In this study, we revealed the complicated roles of miR-320 in nucleus and cytoplasm. Exogenous miR-320 administration increased cytoplasmic miR-320 level and suppressed LEPR, leading to further increased lipogenic enzyme FAS, while in nucleus, miR-320 enhanced CD36 activation and increased fatty acid uptake. Together, nuclear miR-320 and cytosol miR-320 collectively promoted lipid content in hepatic cells by targeting CD36 and leptin receptor respectively. Conversely, miR-320 knockdown decreased lipogenic gene FAS by enhancing LEPR in the cytoplasm and downregulating CD36 transcription in the nucleus, which provided a potential therapeutic target for treating NAFLD in the future.

MiRNAs are in the spotlight as post-transcriptional regulators for gene expression. MiRNAs usually negatively regulate gene translation by affecting mRNA stability in the cytoplasm [21]. However, the roles of miRNAs in subcellular fractions such as nucleus and mitochondria have been revealed recently [16, 17]. In fact, the majority of cellular miRNAs were present in both the nucleus and the cytoplasm [22, 23]. In the nucleus, miRNAs acted to regulate the stability of nuclear transcripts, induce epigenetic alterations that either silence or activate transcription at specific gene promoters [24]. Despite these findings, the comprehensive roles and detailed mechanisms of nuclear miRNAs are largely unknown. Moreover, it’s also unknown why nuclear miR-320 was increased while cytosol miR-320 was decreased in NAFLD mice. Several studies showed that XPO1 (Exportin-1) and IPO8 (Importin-8) facilitated the shuttle of mature miRNAs between the cytoplasm and nucleus [25, 26]. Whether XPO1 and IPO8 were responsible for rearranged subcellular miR-320 localization in NAFLD remain to be determined in the future.

Our previous work has demonstrated that miR-320 is up-regulated in the heart by hyperglycaemia, which acts in the nucleus to enhance CD36 transcription and promote FA uptake, eventually leading to myocardial lipid deposition [14]. Our present work indicated that this is also the case for nuclear miR-320 action in liver, that miR-320 translocated into nucleus to enhance CD36 activation in NAFLD. Inhibition of miR-320 decreased miR-320 level in cytosol, increased leptin receptor translation and decreased de novo lipogenesis. Meanwhile, miR-320 inhibitor also decreased miR-320 level in nucleus, reduced CD36 transcription and lipid uptake, which collectively leading to rescued liver lipid overload. miR-320 might serve as an intriguing subject for future studies to understand the complex and coordinated action of miRNAs in subcellular fractions during multiple diseases.

## MATERIALS AND METHODS

### Biochemical parameters

Circulating Triglyceride (TG), total cholesterol (TC) levels were detected using GRO-PAP method (Nanjing Jiancheng Bioengineering Institute, Nanjing, China). Mice were fasted overnight, then blood glucose levels were measured by Glucose LiquiColor Test (Stanbio Laboratory, Boerne, TX).

Hepatic triglyceride levels were determined using liver homogenate by GRO-PAP method (Nanjing Jiancheng Bioengineering Institute, Nanjing, China) after mice were sacrificed.

### Generation of miR-320 transgenic (tg) mice and in situ hybridization

To generate miR-320 tg mice, a DNA fragment containing murine miR-320 was inserted into the pUBC vector for expression under the control of the ubiquitin C promoter. Microinjection was performed according to standard protocols. miR-320 tg mice were back-crossed into the C57BL/6 background for six generations, yielding wt and miR-320 tg mice that were > 95% of the C57BL/6 genotype. The primers for genotyping miR-320 tg mice were described previously [14].

### STZ treatment

Type 1 diabetes model was established by intraperitoneal STZ (Sigma, St. Louis, MO; dissolved in 100 mM Na-Citrate Buffer, pH 4.5) injection at a dose of 50 mg/kg for 5 consecutive days as described [27, 28]. One week after STZ administration, C57BL/6 mice with fasting blood glucose over 11.1 mmol/L in two consecutive analyses were considered as diabetes mice. Detailed animal procedure was presented in Supplementary Figure 1A, 1D, 1G.

### HFD treatment

8-week old C57BL/6 mice were fed with a control diet (CHOW) or an HFD for 4 weeks until euthanization at 12 weeks of age. HFD contains 20 kcal% protein, 20 kcal% carbohydrate, and 60 kcal% fat. In contrast, control diet contains 20 kcal% protein, 70 kcal% carbohydrate and 10 kcal% fat. HFD and control diet were purchased from Beijing Huafukang Bioscience Co., Ltd. (Beijing, China). Detailed animal procedure was presented in Supplementary Figure 1B, 1E, 1H.

### db/db model

6-week-old male db/db mice (LEPR deficiency) and their wild type littermates (BKS) were purchased from GemPharmatech (Nanjing, China). Detailed animal procedure was presented in Supplementary Figure 1C, 1F, 1I.

### Application of recombinant adeno-associated viruses in animals

Recombinant adeno-associated virus (rAAV) vectors (type 9) were used to express *miR-320, miR-320-TUD (i.e., inhibitor),* or appropriate controls in the liver *in vivo*. The rAAV9-CMV system was a kind gift from Dr. Xiao Xiao (University of North Carolina at Chapel Hill). The three plasmids used to co-transfect HEK293 cells were purified as we described previously [16, 29].

rAAV8-TBG-vector were used to specifically express *miR-320 or miR-320-TUD* into liver. The rAAV8 vectors were constructed by ViGene BioSciences (Shandong, China). pAAV-miR-320 sequence: TCGCCCTCTCAACCCAGCTTTTTTCAAGAGAAAAAGCTGGGTTGAGAGGGCGA, pAAV-miR-320-TUD sequence: GACGGCGCTAGGATCATCAACTCGCCCTCTCAAATCTCCCAGCTTTTCAAGTATTCTGGTCACAGAATACAACTCGCCCTCTCAAATCTCCCAGCTTTTCAAGATGATCCTAGCGCCGTCTTTTTT.

The viruses (at 1×10^12^ vector copy numbers) were delivered into C57BL/6 mice at 6 weeks old via tail vein infection. As it usually required 1-2 weeks for rAAV to express stably, STZ or HFD were administrated to these mice two weeks after rAAV injection (8 weeks old). BKS and db/db mice were also treated with rAAV at 6 weeks old. All animals were sacrificed and detected at 12 weeks of age. Detailed animal procedure was presented in Supplementary Figure 1D–1I.

### Cell culture and transfection

Human hepatic cell line cells (L02) were maintained in RPMI-1640 medium with 10% FBS (Life Technologies, Carlsbad, CA). miRNA mimics (50 nM, AAAAGCUGGGUUGAGAGGGCGA, 5’ to 3’), inhibitors (50 nM, UCGCCCUCUCAACCCAGCUUUU, 5’ to 3’), siRNAs (50 nM), and random small RNA controls (50 nM) were transfected by Lipofectamine 2000 (Life Technologies, Carlsbad, CA). All the small RNAs used in the present study were purchased from Riobio Co., Ltd (Guangzhou, China).

### RNA extraction and quantitative RT-PCR

Total RNA was isolated using TRIzol (Invitrogen, Carlsbad, CA) and reversely transcribed with RevertAid First Strand cDNA Synthesis Kit (Thermo Scientific, Carlsbad, CA). Real-time PCR assays were performed with the SYBR® Select Master Mix (Life Technologies, Carlsbad, CA) on a 7900HT FAST Real-Time PCR System (Life Technologies, Carlsbad, CA). Relative expression levels were calculated with the 2^-ΔΔct^ relative quantification method as described [30].

### Protein extraction and western blotting

The protein concentrations were determined by BCA method. For western blotting, the total cell lysate was resolved by SDS-PAGE, transferred on to nitrocellulose membrane, and blocked with 5% non-fat dry milk in TBS-T. The membrane was incubated with indicated primary antibody overnight at 4 °C, followed by incubation with peroxidase-conjugated secondary antibody for 2 h, and finally developed with the ECL system (Beyotime Institute of Biotechnology, Nanjing, China). The antibodies used in the present study are listed in Supplementary Table 1. The western blotting results were quantified by densitometry and processed with Image J software (National Institutes of Health software).

### Chromatin immunoprecipitation (ChIP)-PCR

ChIP assay was performed to evaluate Ago2 targeting sites at specific promoters. Briefly, the L02 cells were fixed with 1% formaldehyde for 10 min at room temperature and then quenched by adding 125 mM glycine. The samples were homogenized in lysis buffer and sonicated to generate chromatin samples with average fragment sizes of 200-1000 bps. The samples were then incubated with anti-Ago2 (Abnova, Cat# H00027161-M01), or control IgG at 4 °C for overnight in an inverse rotator. Following that, the Pierce™ protein A/G Magnetic Beads (Thermo Fisher Scientific, Cat# 88802) was added to the reaction and gently vortexed. After standard wash, the immunoprecipitated DNA was eluted and purified with PCR Purification Kit. RT-PCR was then performed using primers targeting the promoter regions of the selected genes (Supplementary Table 2).

### RNA immunoprecipitation

Lysed cell extracts were immunoprecipitated with anti-Ago2 antibody or IgG, as described [30]. After elution from the beads, bound RNA was extracted with TRIzol and quantified by real time RT-PCR.

### Subcellular fractionation

The cytoplasmic and nuclear fractions were prepared using a Cell Fractionation Kit (Cell Signalling Technology, Danvers, MA; Cat# 9038) following the manufacturer’s instructions. Real-time PCR analysis was performed using glyceraldehyde 3-phosphate dehydrogenase or U6 as the cytoplasmic and nuclear markers, respectively.

### Histopathology and immunohistochemical staining

The formalin-fixed livers were embedded in paraffin and sectioned into 4 μm slices. The morphology and lipid deposition were detected by Hematoxylin and Eosin (H&E) and Oil red O staining, respectively. The tissue sections were visualized by microscope and quantified by mage-Pro Plus Version 6.0 (Media Cybernetics, Bethesda, MD).

### Statistical analysis

Data are presented as mean ± SEM (n was noted in specific figure legends). Student’s t tests and ANOVA with Bonferroni post hoc analysis were performed to determine statistical significance between different groups. In all cases, statistical significance was defined as p < 0.05.

### Ethics

All animal studies were conducted with the approval of the Animal Research Committee of Tongji Medical College, and in accordance with the NIH Guide for the Care and Use of Laboratory Animals. 6-week-old male C57BL/6 mice were purchased from GemPharmatech (Nanjing, China).

## Supplementary Material

Supplementary Figures

Supplementary Tables

## References

[1] Rinella M, Charlton M. The globalization of nonalcoholic fatty liver disease: prevalence and impact on world health. Hepatology. 2016; 64:19–22. 10.1002/hep.28524 26926530

[2] Younossi ZM, Koenig AB, Abdelatif D, Fazel Y, Henry L, Wymer M. Global epidemiology of nonalcoholic fatty liver disease-meta-analytic assessment of prevalence, incidence, and outcomes. Hepatology. 2016; 64:73–84. 10.1002/hep.28431 26707365

[3] Chalasani N, Younossi Z, Lavine JE, Diehl AM, Brunt EM, Cusi K, Charlton M, Sanyal AJ. The diagnosis and management of non-alcoholic fatty liver disease: practice Guideline by the American Association for the Study of Liver Diseases, American College of Gastroenterology, and the American Gastroenterological Association. Hepatology. 2012; 55:2005–23. 10.1002/hep.25762 22488764

[4] Yilmaz Y, Eren F. Hepatic fibrosis - and not steatosis - is the main determinant of arterial stiffness in non-alcoholic fatty liver disease. Atherosclerosis. 2019; 290:222–23. 10.1016/j.atherosclerosis.2019.07.020 31492444

[5] Musso G, Gambino R, Cassader M, Pagano G. Meta-analysis: natural history of non-alcoholic fatty liver disease (NAFLD) and diagnostic accuracy of non-invasive tests for liver disease severity. Ann Med. 2011; 43:617–49. 10.3109/07853890.2010.518623 21039302

[6] Ortiz-Lopez C, Lomonaco R, Orsak B, Finch J, Chang Z, Kochunov VG, Hardies J, Cusi K. Prevalence of prediabetes and diabetes and metabolic profile of patients with nonalcoholic fatty liver disease (NAFLD). Diabetes Care. 2012; 35:873–78. 10.2337/dc11-1849 22374640 PMC3308295

[7] Xu H, Barnes GT, Yang Q, Tan G, Yang D, Chou CJ, Sole J, Nichols A, Ross JS, Tartaglia LA, Chen H. Chronic inflammation in fat plays a crucial role in the development of obesity-related insulin resistance. J Clin Invest. 2003; 112:1821–30. 10.1172/JCI19451 14679177 PMC296998

[8] Kraegen EW, Clark PW, Jenkins AB, Daley EA, Chisholm DJ, Storlien LH. Development of muscle insulin resistance after liver insulin resistance in high-fat-fed rats. Diabetes. 1991; 40:1397–403. 10.2337/diab.40.11.1397 1936601

[9] Donnelly KL, Smith CI, Schwarzenberg SJ, Jessurun J, Boldt MD, Parks EJ. Sources of fatty acids stored in liver and secreted via lipoproteins in patients with nonalcoholic fatty liver disease. J Clin Invest. 2005; 115:1343–51. 10.1172/JCI23621 15864352 PMC1087172

[10] Anstee QM, Targher G, Day CP. Progression of NAFLD to diabetes mellitus, cardiovascular disease or cirrhosis. Nat Rev Gastroenterol Hepatol. 2013; 10:330–44. 10.1038/nrgastro.2013.41 23507799

[11] Lonardo A, Nascimbeni F, Mantovani A, Targher G. Hypertension, diabetes, atherosclerosis and NASH: cause or consequence? J Hepatol. 2018; 68:335–52. 10.1016/j.jhep.2017.09.021 29122390

[12] Hebbard L, George J. Animal models of nonalcoholic fatty liver disease. Nat Rev Gastroenterol Hepatol. 2011; 8:35–44. 10.1038/nrgastro.2010.191 21119613

[13] Chen C, Wang Y, Yang S, Li H, Zhao G, Wang F, Yang L, Wang DW. MiR-320a contributes to atherogenesis by augmenting multiple risk factors and down-regulating SRF. J Cell Mol Med. 2015; 19:970–85. 10.1111/jcmm.12483 25728840 PMC4420600

[14] Li H, Fan J, Zhao Y, Zhang X, Dai B, Zhan J, Yin Z, Nie X, Fu XD, Chen C, Wang DW. Nuclear miR-320 mediates diabetes-induced cardiac dysfunction by activating transcription of fatty acid metabolic genes to cause lipotoxicity in the heart. Circ Res. 2019; 125:1106–20. 10.1161/CIRCRESAHA.119.314898 31638474 PMC6903355

[15] Ti Y, Xie GL, Wang ZH, Bi XL, Ding WY, Wang J, Jiang GH, Bu PL, Zhang Y, Zhong M, Zhang W. TRB3 gene silencing alleviates diabetic cardiomyopathy in a type 2 diabetic rat model. Diabetes. 2011; 60:2963–74. 10.2337/db11-0549 21933987 PMC3198078

[16] Li H, Dai B, Fan J, Chen C, Nie X, Yin Z, Zhao Y, Zhang X, Wang DW. The different roles of miRNA-92a-2-5p and let-7b-5p in mitochondrial translation in db/db mice. Mol Ther Nucleic Acids. 2019; 17:424–35. 10.1016/j.omtn.2019.06.013 31319246 PMC6637210

[17] Zhang X, Zuo X, Yang B, Li Z, Xue Y, Zhou Y, Huang J, Zhao X, Zhou J, Yan Y, Zhang H, Guo P, Sun H, et al. MicroRNA directly enhances mitochondrial translation during muscle differentiation. Cell. 2014; 158:607–19. 10.1016/j.cell.2014.05.047 25083871 PMC4119298

[18] Sharma NR, Wang X, Majerciak V, Ajiro M, Kruhlak M, Meyers C, Zheng ZM. Cell type- and tissue context-dependent nuclear distribution of human Ago2. J Biol Chem. 2016; 291:2302–09. 10.1074/jbc.C115.695049 26699195 PMC4732213

[19] Tamura S, Shimomura I. Contribution of adipose tissue and de novo lipogenesis to nonalcoholic fatty liver disease. J Clin Invest. 2005; 115:1139–42. 10.1172/JCI24930 15864343 PMC1087181

[20] Kim TT, Dyck JR. The role of CD36 in the regulation of myocardial lipid metabolism. Biochim Biophys Acta. 2016; 1861:1450–60. 10.1016/j.bbalip.2016.03.018 26995462

[21] Wang F, Fang Q, Chen C, Zhou L, Li H, Yin Z, Wang Y, Zhao CX, Xiao X, Wang DW. Recombinant adeno-associated virus-mediated delivery of MicroRNA-21-3p lowers hypertension. Mol Ther Nucleic Acids. 2018; 11:354–66. 10.1016/j.omtn.2017.11.007 29858071 PMC5992325

[22] Khudayberdiev SA, Zampa F, Rajman M, Schratt G. A comprehensive characterization of the nuclear microRNA repertoire of post-mitotic neurons. Front Mol Neurosci. 2013; 6:43. 10.3389/fnmol.2013.00043 24324399 PMC3840315

[23] Liao JY, Ma LM, Guo YH, Zhang YC, Zhou H, Shao P, Chen YQ, Qu LH. Deep sequencing of human nuclear and cytoplasmic small RNAs reveals an unexpectedly complex subcellular distribution of miRNAs and tRNA 3’ trailers. PLoS One. 2010; 5:e10563. 10.1371/journal.pone.0010563 20498841 PMC2871053

[24] Roberts TC. The MicroRNA biology of the mammalian nucleus. Mol Ther Nucleic Acids. 2014; 3:e188. 10.1038/mtna.2014.40 25137140 PMC4221600

[25] Castanotto D, Lingeman R, Riggs AD, Rossi JJ. CRM1 mediates nuclear-cytoplasmic shuttling of mature microRNAs. Proc Natl Acad Sci USA. 2009; 106:21655–59. 10.1073/pnas.0912384106 19955415 PMC2787469

[26] Weinmann L, Höck J, Ivacevic T, Ohrt T, Mütze J, Schwille P, Kremmer E, Benes V, Urlaub H, Meister G. Importin 8 is a gene silencing factor that targets argonaute proteins to distinct mRNAs. Cell. 2009; 136:496–507. 10.1016/j.cell.2008.12.023 19167051

[27] Chaudhry ZZ, Morris DL, Moss DR, Sims EK, Chiong Y, Kono T, Evans-Molina C. Streptozotocin is equally diabetogenic whether administered to fed or fasted mice. Lab Anim. 2013; 47:257–65. 10.1177/0023677213489548 23760565 PMC3773005

[28] Mishra AP, Yedella K, Lakshmi JB, Siva AB. Wdr13 and streptozotocin-induced diabetes. Nutr Diabetes. 2018; 8:57. 10.1038/s41387-018-0065-6 30369599 PMC6204428

[29] Dai B, Li H, Fan J, Zhao Y, Yin Z, Nie X, Wang DW, Chen C. MiR-21 protected against diabetic cardiomyopathy induced diastolic dysfunction by targeting gelsolin. Cardiovasc Diabetol. 2018; 17:123. 10.1186/s12933-018-0767-z 30180843 PMC6122727

[30] Li H, Zhang X, Wang F, Zhou L, Yin Z, Fan J, Nie X, Wang P, Fu XD, Chen C, Wang DW. MicroRNA-21 lowers blood pressure in spontaneous hypertensive rats by upregulating mitochondrial translation. Circulation. 2016; 134:734–51. 10.1161/CIRCULATIONAHA.116.023926 27542393 PMC5515592

