# Supplementary material for: The nuclear and cytoplasmic roles of miR-320 in non-alcoholic fatty liver disease
Source: Aging (Albany NY). 2020 Nov 7;12(21):22019–45. doi: 10.18632/aging.104040 (PMC11623971; doi:10.18632/aging.104040)
Supplement: Supplementary Figures [file aging-12-104040-s001.pdf]

SUPPLEMENTARY FIGURES

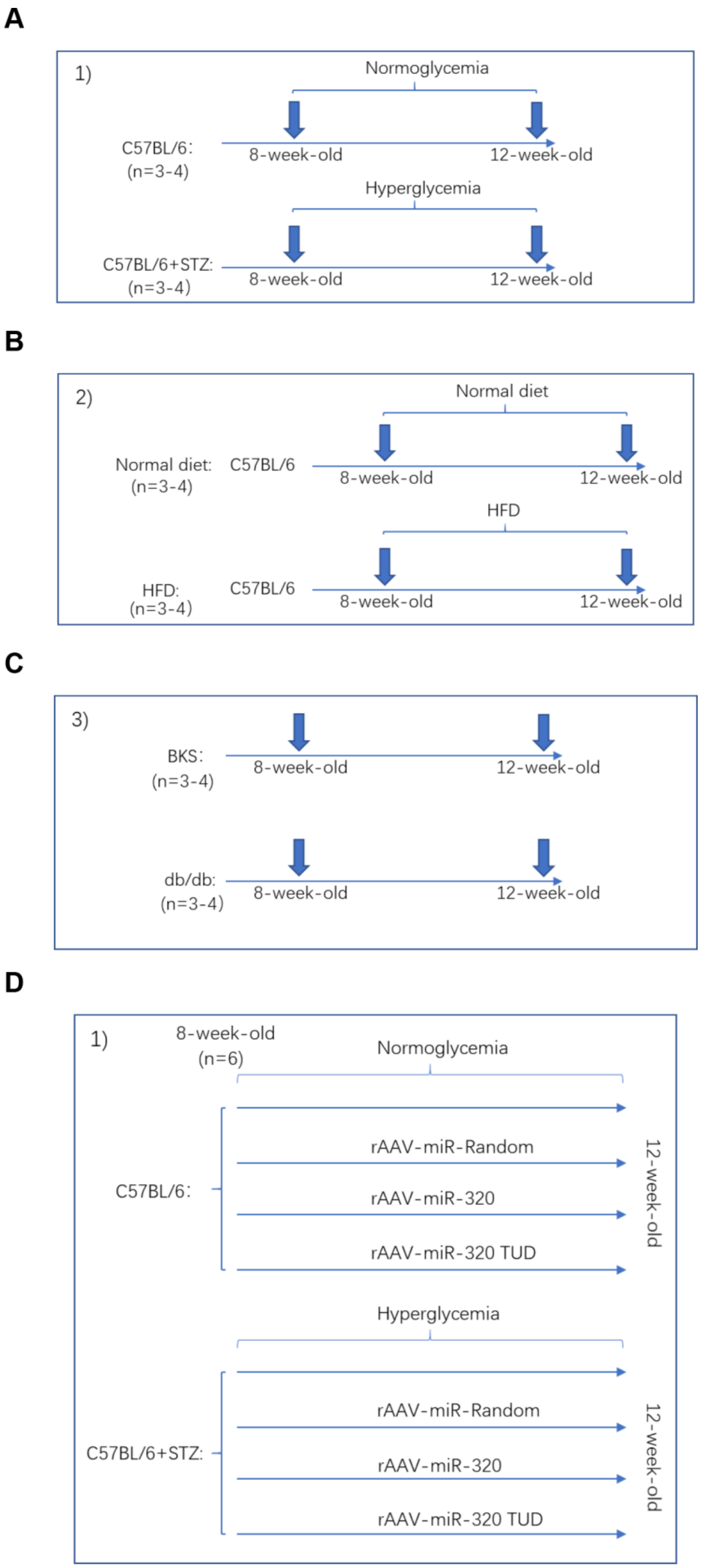

E

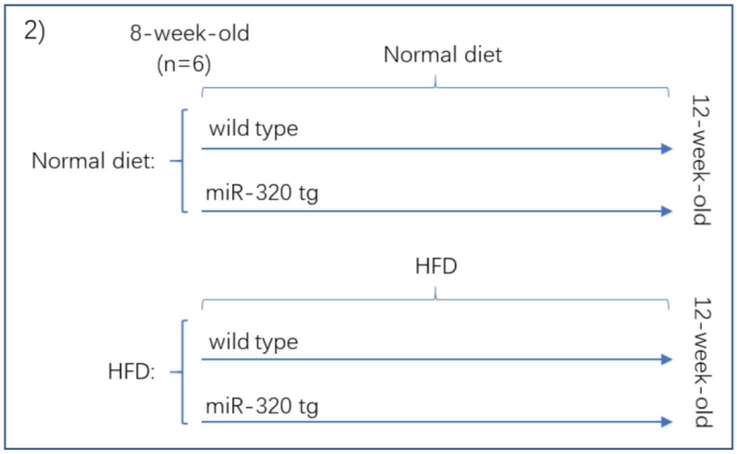
2)
8-week-old
(n=6)
Normal diet
Normal diet:
wild type
miR-320 tg
HFD
HFD:
wild type
miR-320 tg
12-week-old
12-week-old

F

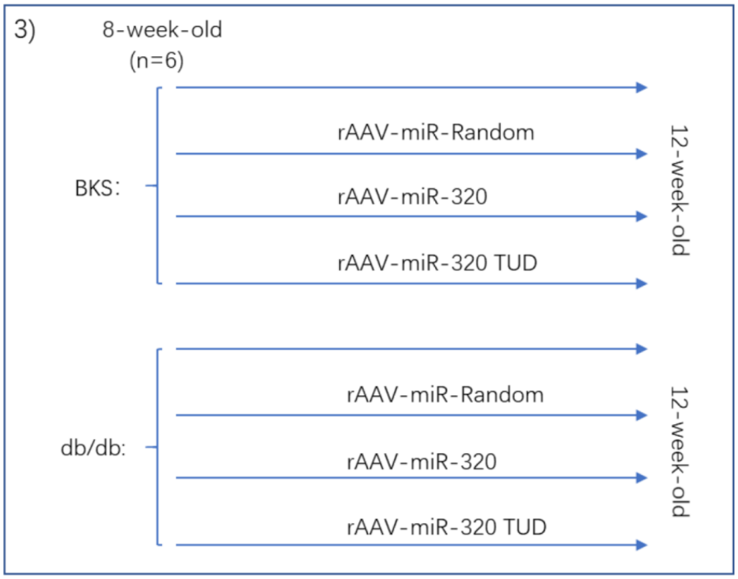
3)
8-week-old
(n=6)
BKS:
rAAV-miR-Random
rAAV-miR-320
rAAV-miR-320 TUD
db/db:
rAAV-miR-Random
rAAV-miR-320
rAAV-miR-320 TUD
12-week-old
12-week-old

G

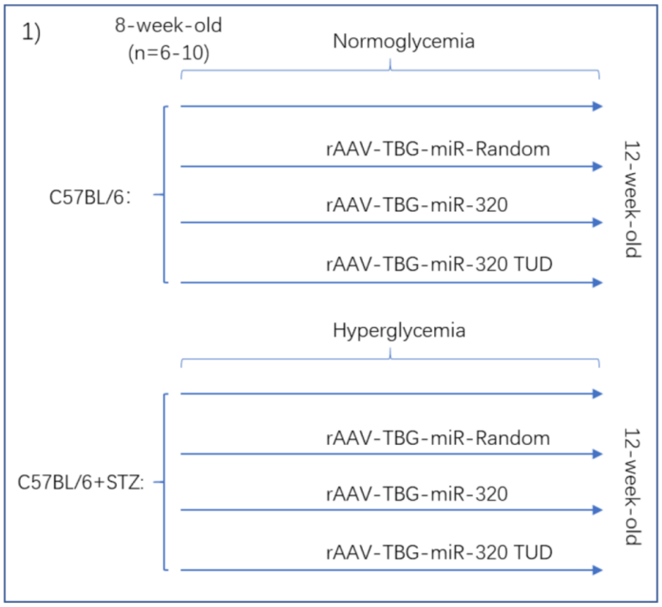
1)
8-week-old
(n=6-10)
Normoglycemia
C57BL/6:
rAAV-TBG-miR-Random
rAAV-TBG-miR-320
rAAV-TBG-miR-320 TUD
Hyperglycemia
C57BL/6+STZ:
rAAV-TBG-miR-Random
rAAV-TBG-miR-320
rAAV-TBG-miR-320 TUD
12-week-old
12-week-old

H

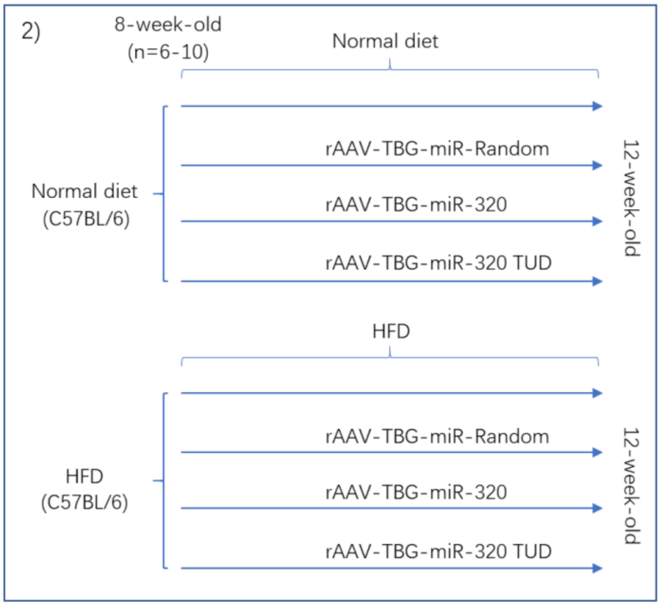

I

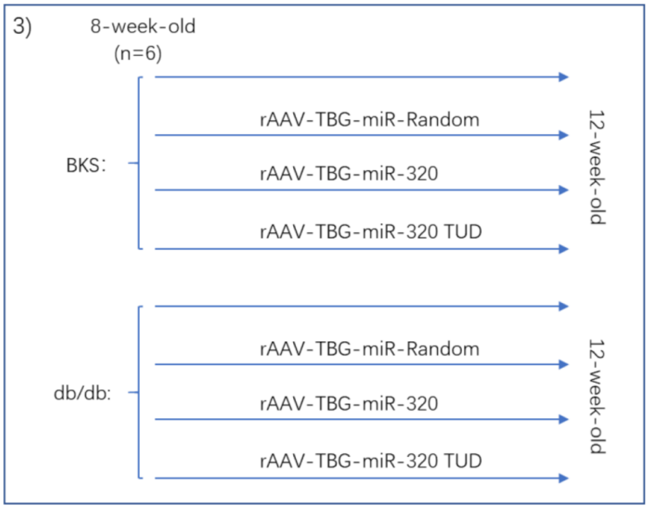

Supplementary Figure 1. The different animal treatment strategies. (**A**–**C**) The effects of single metabolic disorder on NAFLD. (**D**–**F**) The effects of miR-320 on liver lipid content by rAAV9 vector in differently treated mice. (**G**–**I**) The effects of miR-320 on liver lipid content by AAV8-TBG vector in differently treated mice.

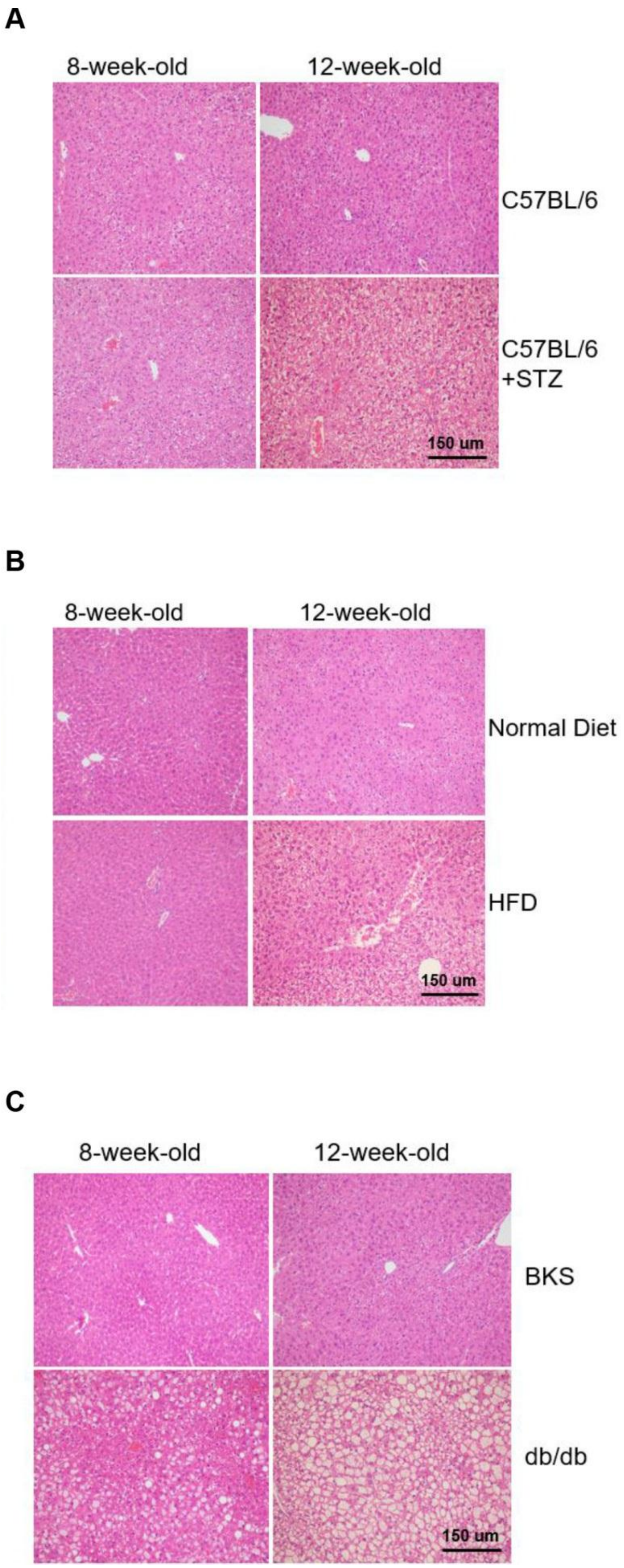

Supplementary Figure 2. HE staining in liver of differently treated mice. (**A**–**C**) Representative image of liver steatosis detected by HE staining in STZ-treated C57BL/6 mice (**A**), HFD-treated C57BL/6 mice (**B**) and db/db mice (**C**) at different time point.

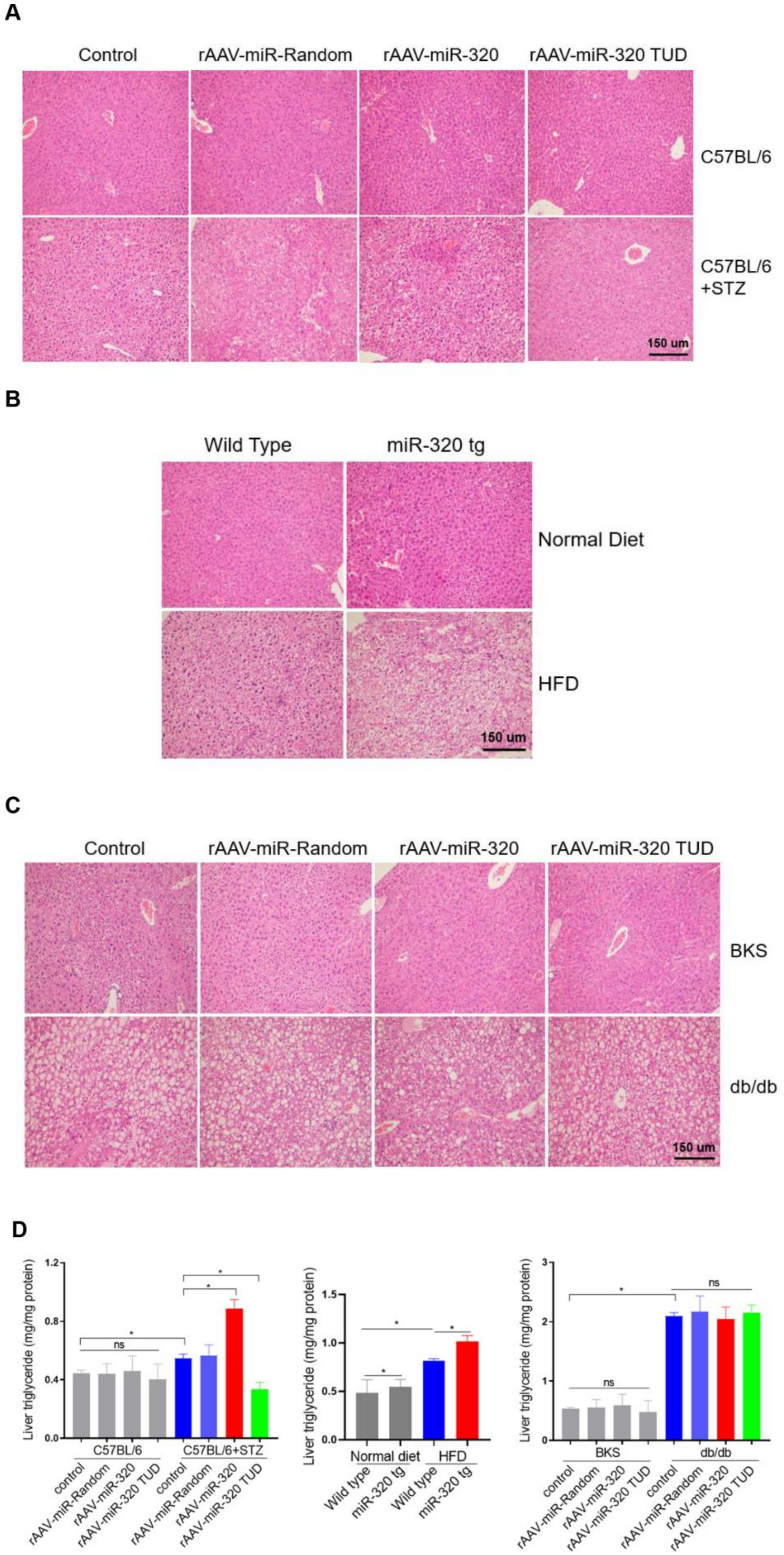

Supplementary Figure 3. MiR-320 overexpression increased hepatic steatosis and lipid content in differently treated mice. (**A**) Representative image of hepatic H&E staining in STZ-treated C57BL/6 mice with various rAAV9 treatments. (**B**) Representative image of hepatic H&E staining in miR-320 transgenic mice treated with normal diet or HFD. (**C**) Representative image of hepatic H&E staining in db/db mice and BKS controls with various rAAV9 treatments. (**D**) Hepatic triglyceride levels in these three animal models (n=3-5, *p<0.05).

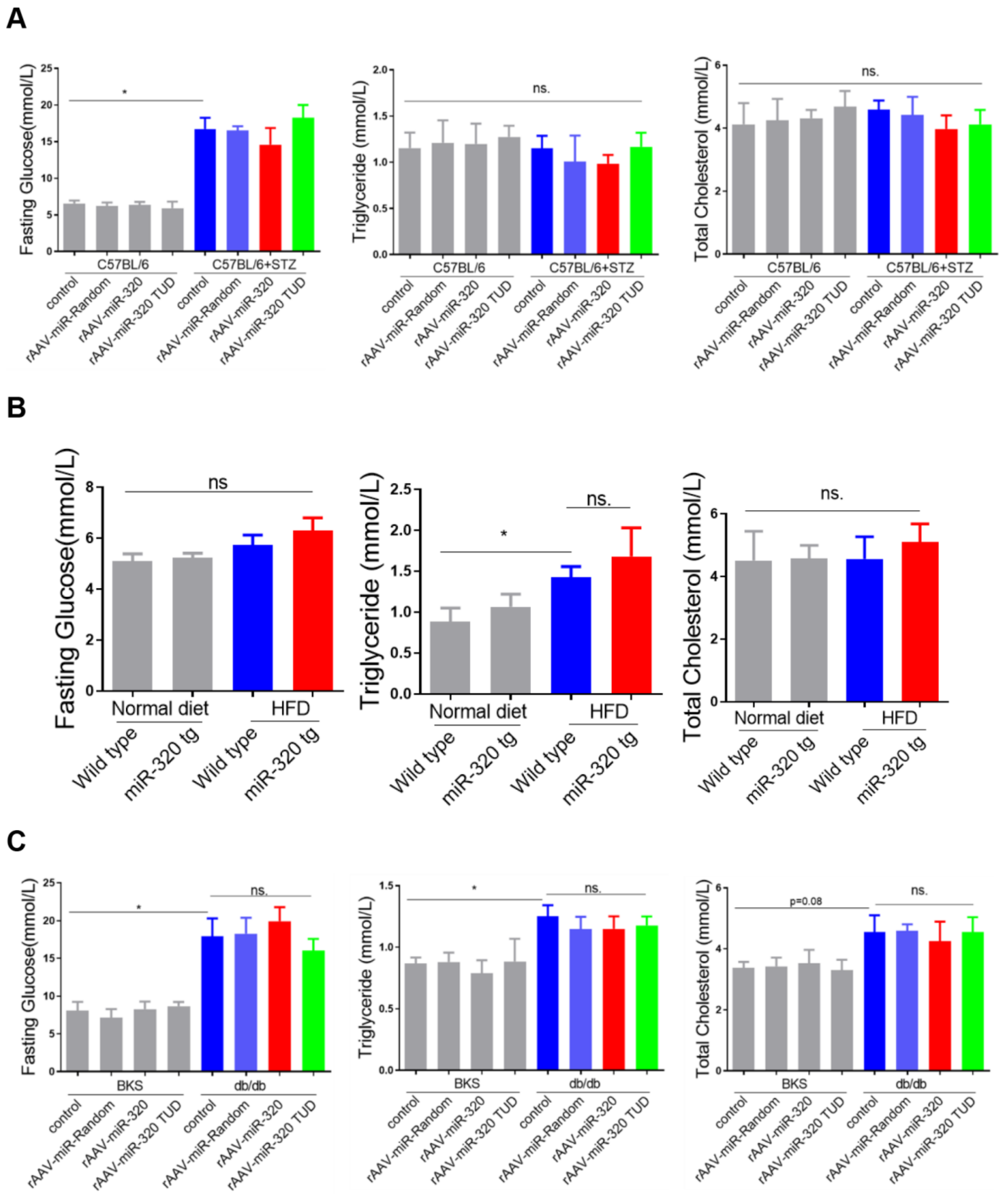

Supplementary Figure 4. Circulating glucose, TC and TG levels in differently treated mice. (**A**) Circulating glucose, TC and TG levels in STZ-induced C57BL/6 mice treated with rAAV-miR-320 (n=4-5, *p<0.05). (**B**) Circulating glucose, TC and TG levels in miR-320 transgenic mice treated with high fat diet (n=4-5, *p<0.05). (**C**) Circulating glucose, TC and TG levels in db/db mice treated with rAAV-miR-320 (n=4-5, *p<0.05).

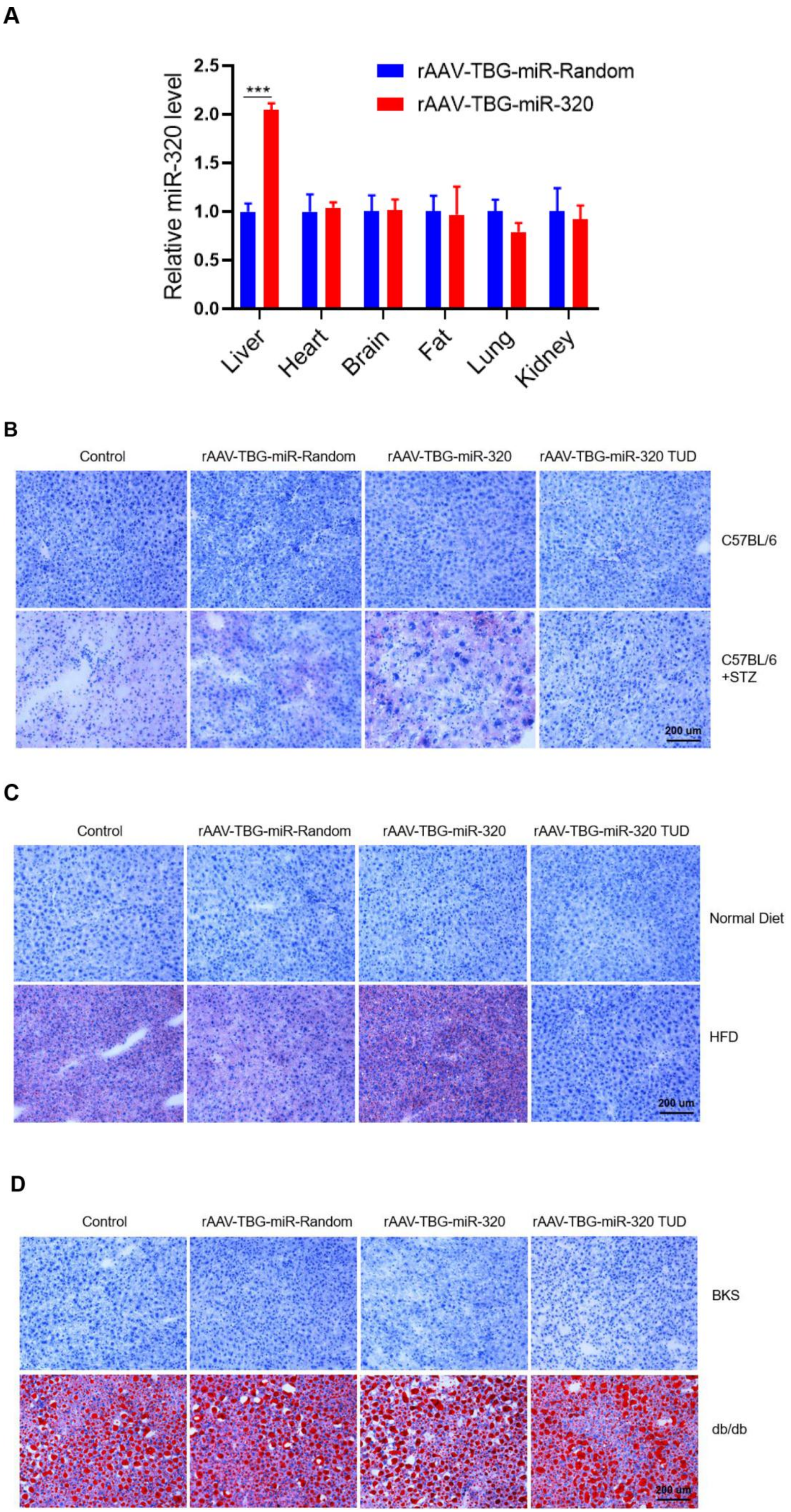
A
rAAV-TBG-miR-Random
rAAV-TBG-miR-320
Relative miR-320 level
2.5
2.0
1.5
1.0
0.5
0.0
***
Liver
Heart
Brain
Fat
Lung
Kidney
B
Control
rAAV-TBG-miR-Random
rAAV-TBG-miR-320
rAAV-TBG-miR-320 TUD
C57BL/6
C57BL/6
+STZ
200 um
C
Control
rAAV-TBG-miR-Random
rAAV-TBG-miR-320
rAAV-TBG-miR-320 TUD
Normal Diet
HFD
200 um
D
Control
rAAV-TBG-miR-Random
rAAV-TBG-miR-320
rAAV-TBG-miR-320 TUD
BKS
db/db
200 um

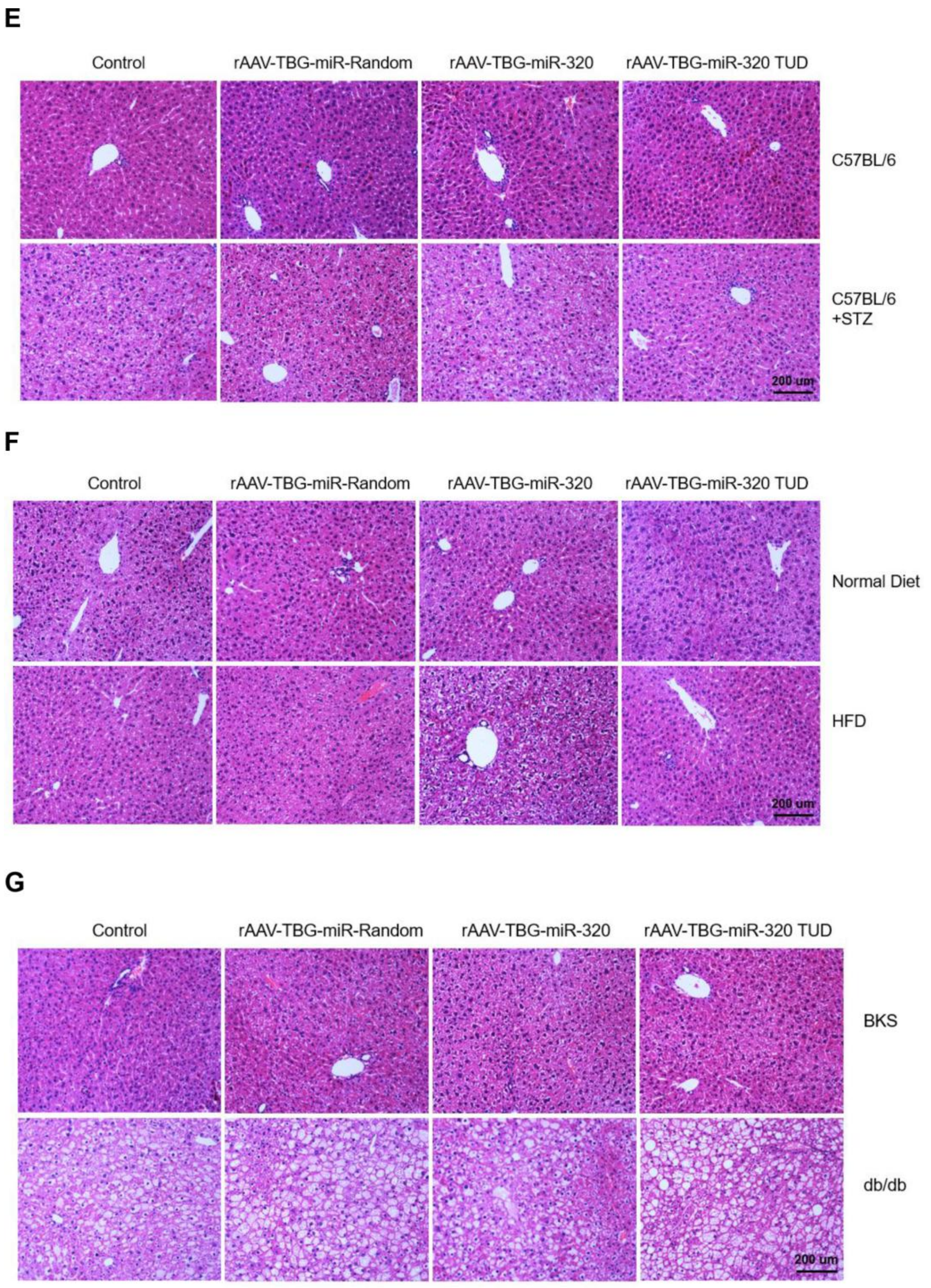

Supplementary Figure 5. MiR-320 overexpression by AAV-TBG vectors increased liver lipid content in differently treated mice. AAV8-TBG vectors were purchased from Vigene-Bioscience (Shangdong, China), and delivered to mice via tail vein injection. (**A**) Relative miR-320 levels of various organs in AAV8-TBG-miR-320 treated mice (n=3-4, *p<0.05). (**B**–**D**) The effects of rAAV8-TBG-miR-320 on lipid accumulation in liver determined by oil red staining in STZ-treated C57BL/6 mice (**B**), HFD-treated C57BL/6 mice (**C**), and db/db mice(**D**). (**E**–**G**) Representive images of hepatic H&E staining in STZ-treated C57BL/6 mice (**E**), HFD-treated C57BL/6 mice (**F**), and db/db mice (**G**) delivered with rAAV8-TBG-miR-320.

A

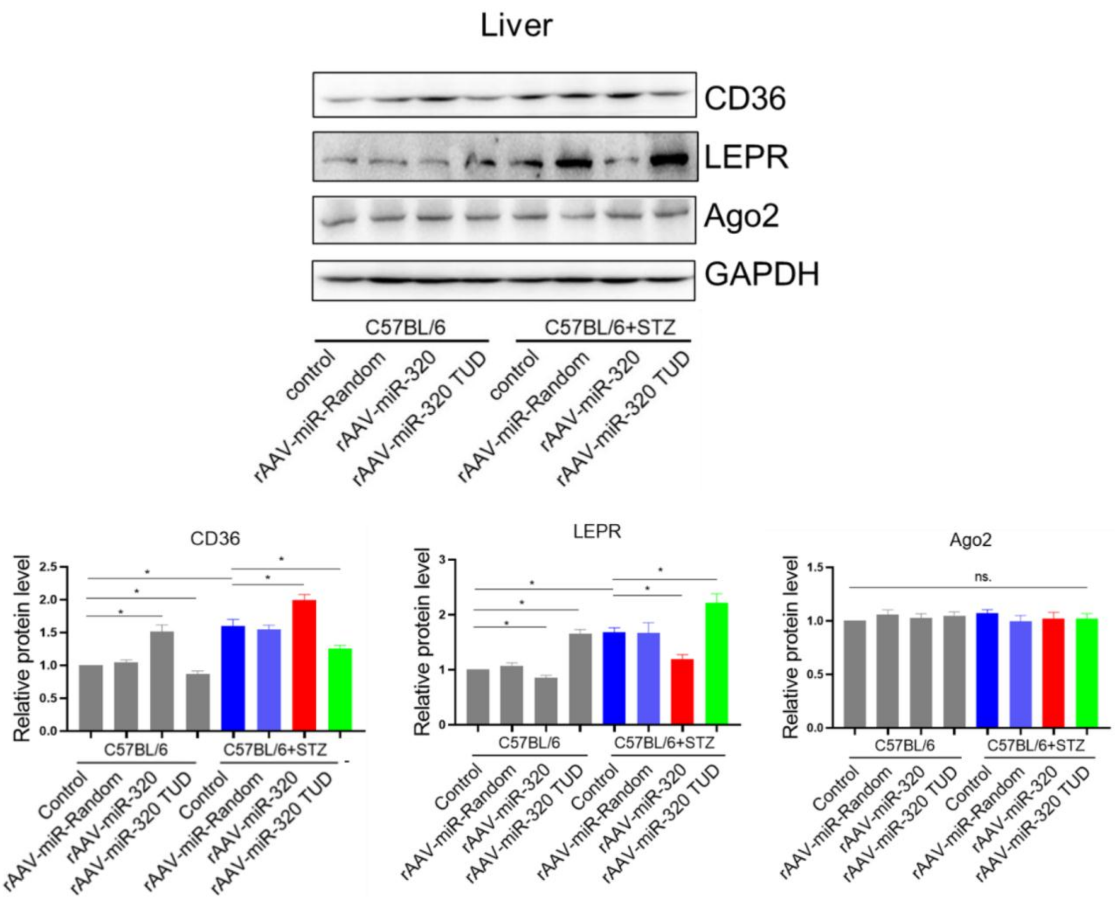
Liver
CD36
LEPR
Ago2
GAPDH
C57BL/6
C57BL/6+STZ
control
rAAV-miR-Random
rAAV-miR-320
rAAV-miR-320 TUD
CD36
LEPR
Ago2
Relative protein level
Control
ns.

B

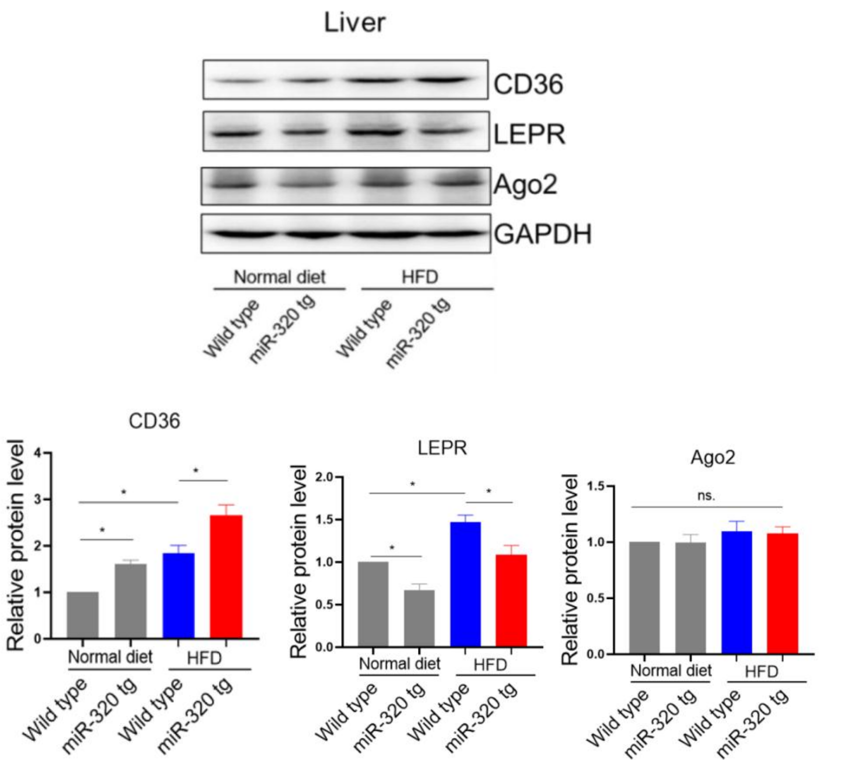
Liver
CD36
LEPR
Ago2
GAPDH
Normal diet
HFD
Wild type
miR-320 tg
Relative protein level
ns.

C

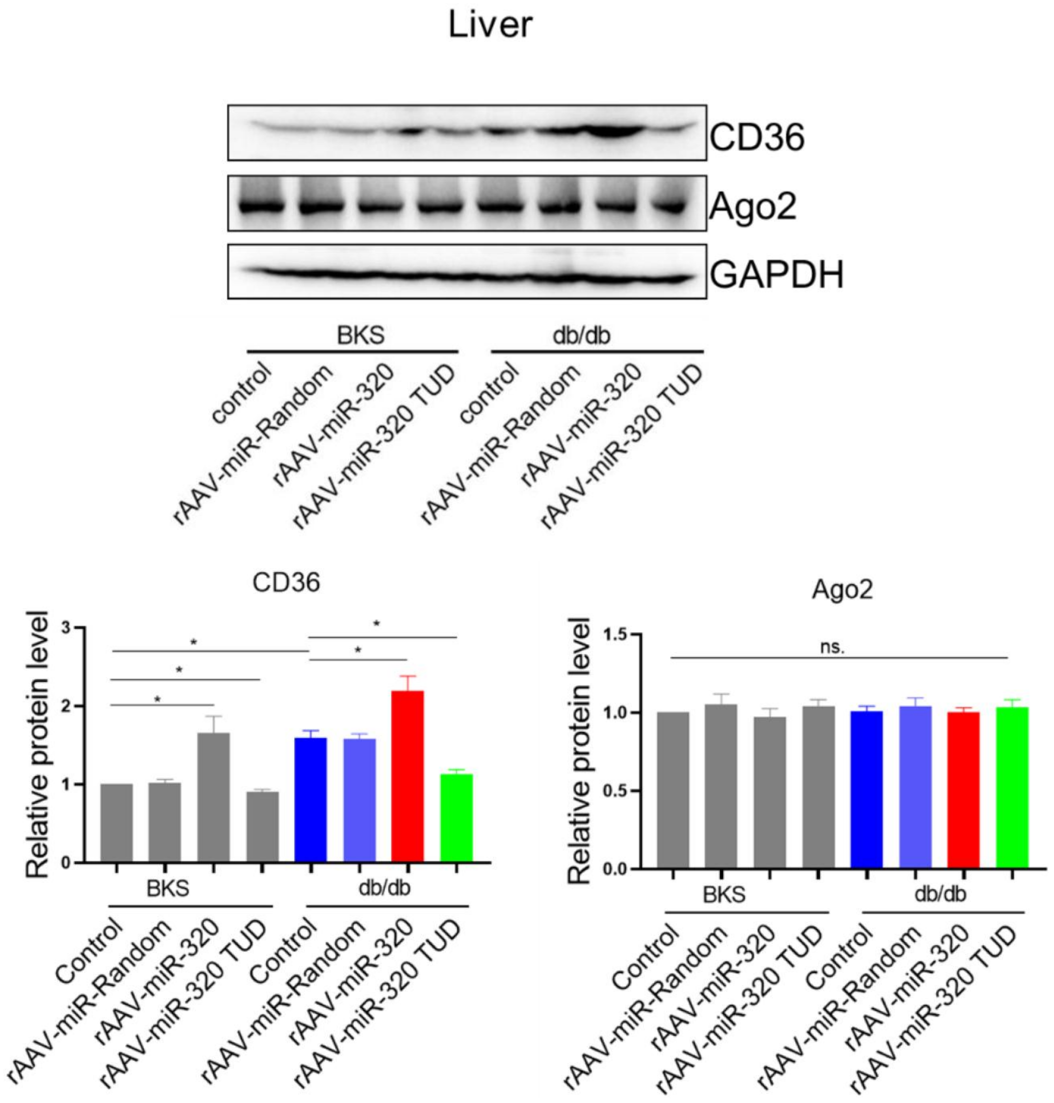

Supplementary Figure 6. miR-320 overexpression upreguleated hepatic CD36 while reduced LEPR protein levels in vivo. (**A**) The effect of rAAV9-miR-320 on the hepatic protein levels of CD36, LEPR and Ago2 in STZ-treated C57BL/6 mice (n=3, *p<0.05). (**B**) Hepatic protein levels of CD36, LEPR and Ago2 in miR-320 transgenic mice treated with HFD (n=3, *p<0.05). (**C**) The effect of rAAV9-miR-320 on the hepatic protein levels of CD36, and Ago2 in db/db mice (n=3, *p<0.05).
