# Supplementary material for: The nuclear and cytoplasmic roles of miR-320 in non-alcoholic fatty liver disease
Source: Aging (Albany NY). 2020 Nov 7;12(21):22019–45. doi: 10.18632/aging.104040 (PMC11623971; doi:10.18632/aging.104040)
Supplement: Supplementary Tables [file aging-12-104040-s002.pdf]

## SUPPLEMENTARY TABLES

**Supplementary Table 1. List of antibodies.**

| Antibody | Company | Catalog number |
|---|---|---|
| AGO2 | Abnova | H00027161-M01 |
| ACC1 | ABclonal | A15606 |
| FASN | ABclonal | A0461 |
| CD36 | ABclonal | A5792 |
| DGAT1 | ABclonal | A6857 |
| DGAT2 | ABclonal | A13891 |
| LEPR | Boster | BA1234 |
| LEP | Boster | BA1231 |
| GAPDH | Boster | BM1623 |

**Supplementary Table 2. List of primers.**

| | Forward 5'→3' | Reverse 5'→3' |
|---|---|---|
| m-SLC27A1 | TCAACAGCCGTATCCTCACG | GGCTCCATCGTGTCCTCATT |
| m-SLC29A2 | GCTGGAGGAAAGACCCAGAC | CACGGTCGATCATCAGGAGG |
| m-CD36 | TGATACTATGCCCGCCTCTCC | TTTCCCACACTCCTTTCTCCTCTA |
| m-FABP1 | AGGGGGTGTCAGAAATCGTG | CCCCCAGGGTGAACTCATTG |
| m-ACC1 | TGGTGAAGCTGGACCTAGAAG | CCTGTAAGCCAGAGATCCCC |
| m-FAS | AGGCCCCTCTGTTAATTGGC | CCCCATGCTCCAGGGATAAC |
| m-DGAT1 | GTTTCCGTCCAGGGTGGTAG | TGGCACCTCAGATCCCAGTA |
| m-DGAT2 | ATTTGGAAGCGTCATGGGTG | CTCCACCTTGAGCAGGACAC |
| m-HMGCS2 | GGATCGATGCTATGCAGCCT | TCAACCGAGCCAGGGATTTC |
| m-CPT2 | CACAGCATCGTACCCACCAT | TCCTTCCCAATGCCGTTCTC |
| m-CPT1A | ACGTTGGACGAATCGGAACA | CCATGCAGCAGAGATTTGGC |
| m-GAPDH | GGTGAAGGTCGGTGTGAACG | CTCGCTCCTGGAAGATGGTG |
| h-LEPR | TGCCTGCTGGACTCTCAAAG | TGCTCACTCCGAAAGCAACA |
| h-GAPDH | CAATGACCCCTTCATTGACC | GACAAGCTTCCCGTTCTCAG |
| h-LEPR-pro | CTTCGAGTAGCGGTAGCGAG | CCTGATAGTTCAGACCCGGC |
| h-CD36-pro | GGAAACTGACTCAAATACACGGA | CAGCTGAGACCACACTCTCAA |
| h-GAPDH-pro | CATTAAGAGGGCGAATGCAGC | CGTATGACTGGGGGTGTTGG |

m, mouse; h, human
